# Continuous‐Flow Synthesis and Derivatization of Aziridines through Palladium‐Catalyzed C(sp^3^)−H Activation

**DOI:** 10.1002/anie.201602483

**Published:** 2016-06-15

**Authors:** Jacek Zakrzewski, Adam P. Smalley, Mikhail A. Kabeshov, Matthew J. Gaunt, Alexei A. Lapkin

**Affiliations:** ^1^ Department of Chemical Engineering and BiotechnologyUniversity of CambridgePembroke StreetCambridgeCB2 3RAUK; ^2^ Chemistry DepartmentUniversity of CambridgeLensfield RdCambridgeCB2 1EWUK

**Keywords:** aziridines, C−H activation, continuous-flow synthesis, palladium catalysis, ring opening

## Abstract

A continuous‐flow synthesis of aziridines by palladium‐catalyzed C(sp^3^)−H activation is described. The new flow reaction could be combined with an aziridine‐ring‐opening reaction to give highly functionalized aliphatic amines through a consecutive process. A predictive mechanistic model was developed and used to design the C−H activation flow process and illustrates an approach towards first‐principles design based on novel catalytic reactions.

Continuous flow processes represent a paradigm shift in the manufacture of fine chemicals, specialties, and pharmaceuticals owing to the demonstrable gains in efficiency and product quality.^1^ A crucial aspect of realizing this ideal is the effective crossover from fundamental chemistry advances to process engineering. It is noticeable that the transition of novel catalytic transformations of potential industrial interest into continuous flow processes is often slow owing to the inherent complexity of the reaction systems. With often limited mechanistic understanding of new reactions, it is rarely straightforward to determine the best reactor configuration or predict their behavior at scale, which can make the design of an optimal process difficult regardless of whether it is batch, semibatch, or continuous. The ultimate desired target for process chemistry is a fully predictive process model that accounts for a wide range of operating conditions and scale effects. However, the development of such models for complex reactions represents a significant methodological challenge.^2^, ^3^

A central field in modern synthetic organic chemistry is transition‐metal‐catalyzed C−H activation. The last 15 years have seen tremendous advances in the use of many different metal catalysts to functionalize traditionally unreactive C−H bonds; palladium salts, in particular, have enjoyed a great deal of success in effecting C−H activation reactions.^4^ Despite the potential of these seemingly ideal strategic bond‐forming reactions, the uptake of C−H activation in pharmaceutical and agrochemical processes and manufacture is limited to a relatively small number of examples.^5^ Part of the reason for this deficiency is the limited mechanistic understanding of these complex reactions, which frequently are heterogeneous under operating conditions; this characteristic can preclude industrial applications of either batch or continuous C−H activation processes.

Recently, one of our groups reported a new palladium‐catalyzed C(sp^3^)−H activation reaction on hindered aliphatic amines to give aziridines (Scheme 1 a).^6^ Having performed some initial mechanistic studies on this transformation,^7^ we considered the possibility of utilizing this reaction as a platform for a C−H activation reaction in flow. Given the prevalence of aliphatic amines in biologically active molecules, a flow‐based C(sp^3^)−H activation process on these molecules would represent an important advance. There are very few examples of flow processes for C(sp^3^)−H activation,^8^ and no flow C(sp^3^)−H activation reactions have been conducted on a gram scale.

**Scheme 1:**
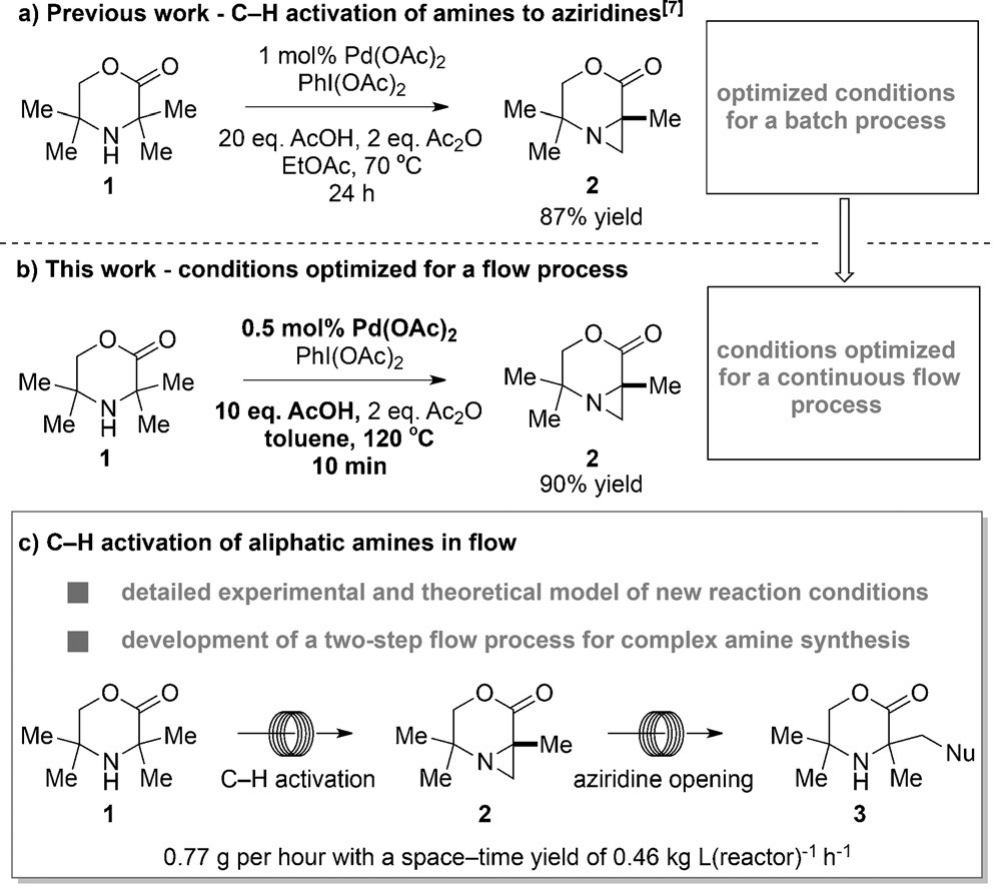
Towards a C−H activation process in flow.

Herein we report the successful translation of first‐principles mechanistic understanding of a C(sp^3^)−H activation reaction for the formation of aziridines into a practical flow process. A predictive process model was used to explore in silico the behavior of a semibatch and a flow reactor prior to testing of the reactors at microreactor and gram scales (Scheme 1 b–c). Additionally, we show that the aziridine products can be used directly as part of a sequential flow process, thus enabling the rapid synthesis of complex aliphatic amines with minimal purification.

During our previous studies, we discovered that the addition of acetic acid to the reaction mixture for the palladium‐catalyzed C−H activation to form aziridines resulted in a significant rate enhancement.^7^ We chose these conditions from which to develop a predictive kinetic model that would facilitate the transfer from batch to flow processes. A proposed catalytic cycle for the C−H activation, accounting for the influence of acetic acid, is shown in Figure 1 a. We began our studies by examining this pathway by quantum‐mechanical and kinetic approaches.

**Figure 1:**
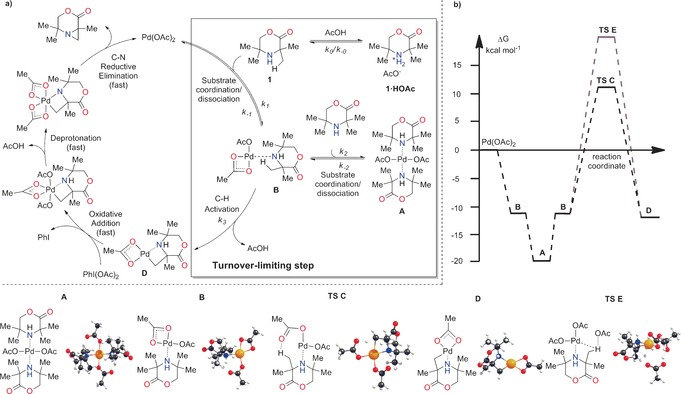
a) Working model for the reaction mechanism. Kinetic parameters corresponding to the reactions preceding the rate‐limiting step are marked “*k*”. The region in the box illustrates the difference between the mechanism under the original reaction conditions and the mechanism under the new fully optimized conditions described herein. b) Energy profile of cyclopalladation proceeding through a CMD mechanism. Values of the kinetic parameters for equilibria at 70 °C: *K*_0_=3.14 L mol^−1^ min^−1^, *K*_1_=2963 160.29 L mol^−1^ min^−1^, *K*_2_=7415.93 L mol^−1^ min^−1^; and for the turnover‐limiting step (70 °C): *k*_3_=9.23 min^−1^.

We focused on the turnover‐limiting step, which was believed to be C−H bond cleavage. This assumption was supported by experimental kinetic isotope effect (KIE) studies,^7^ which gave a value of 3.9. Calculations performed at the density functional theory (DFT) level by the use of Gaussian 09 (Figure 1 b) showed that the bis(amine) complex **A** was the catalyst resting state, as it was thermodynamically the most stable species in the reaction mixture.^9^ The key reactive intermediate (mono(amine)–Pd complex **B**) was found to be less stable by 6.2 kcal mol^−1^. Cyclopalladation from the (mono)amine–Pd complex **B** to form intermediate **D** was characterized as a single elementary step (through concerted metalation–deprotonation, CMD)^10^ with a barrier of 22.9 kcal mol^−1^. We also considered that the higher acetate concentration resulting from the addition of acetic acid may lead to C−H bond cleavage involving an external acetate ligand. However, we found that this pathway was kinetically unfavorable (Gibbs activation energy 38 kcal mol^−1^; **TS** **E**). Instead, we found that protonation of the amine **1** with acetic acid to give **1⋅HOAc** was energetically beneficial by 0.8 kcal mol^−1^. Therefore, we believe that acetic acid protonates the free amine **1** to give **1⋅HOAc**, thereby reducing the free‐amine concentration, which in turn reduces the concentration of the off‐cycle bis(amine)–Pd complex **A**. Thus, palladium is maintained in the catalytic cycle instead of being held in the off‐cycle complex **A**.

Therefore, the mechanism of the acid‐accelerated C−H activation reaction was reduced to a kinetic model incorporating three equilibria (*K*_0_, *K*_1_, and *K*_2_) and one irreversible reaction (*k*_3_; Figure 1 a). The elementary steps following the turnover‐limiting step were ignored in setting up the model. A set of unique, kinetically controlled experiments was designed and performed to build a dataset for parameter estimation. Values of kinetic parameters for equilibria at 70 °C were found to be *K*_0_=3.14 [L mol^−1^ min^−1^], *K*_1_=2963 160.29 [L mol^−1^ min^−1^], *K*_2_=7415.93 [L mol^−1^ min^−1^], and for the turnover‐limiting step (70 °C), *k*_3_=9.23 [min^−1^]. Kinetic parameters were fitted to the experimental data using gPROMS ModelBuilder. The values obtained from parameter estimation are in very good agreement with the values predicted by DFT calculations (see the Supporting Information). The weighted residual was found to be smaller than the *χ* ^2^ value corresponding to the 95 % confidence region, thus indicating that the mechanism shown in Figure 1 a is a good representation of the reaction pathway. Small correlations between most of the kinetic parameters (see the Supporting Information) proved their independence. The larger correlation between *K*_0_ and K_2_ can be explained by their competitive nature with respect to the starting material.

A fully predictive kinetic model allows the selection of optimal conditions to maximize conversion and yield as well as minimize by‐product formation, with a minimum of experimental work. The model also helps to predict the behavior of the reaction system on previously untested scales. All of these factors can result in the faster design of a continuous process. Before transition to the flow setup, we further tested the kinetic model to assess its accuracy under dynamic conditions of a semibatch process. We found that the kinetic model accurately predicted the results for two different semibatch experiments, in which a batch reactor was fed continuously with the starting material, and ideal mass and energy transfer were assumed (Figure 2). A slight deviation of the model from the experimental data was observed in the early stages of the experiment. This deviation can be explained by the fact that the semibatch reactions were performed without preheating of the reaction mixture, and subsequently the reactor operated at lower temperature until the system reached thermal equilibrium. The good agreement between the experimental data and the predicted kinetics provides further support for the model as an accurate representation of the molecular processes.

**Figure 2:**
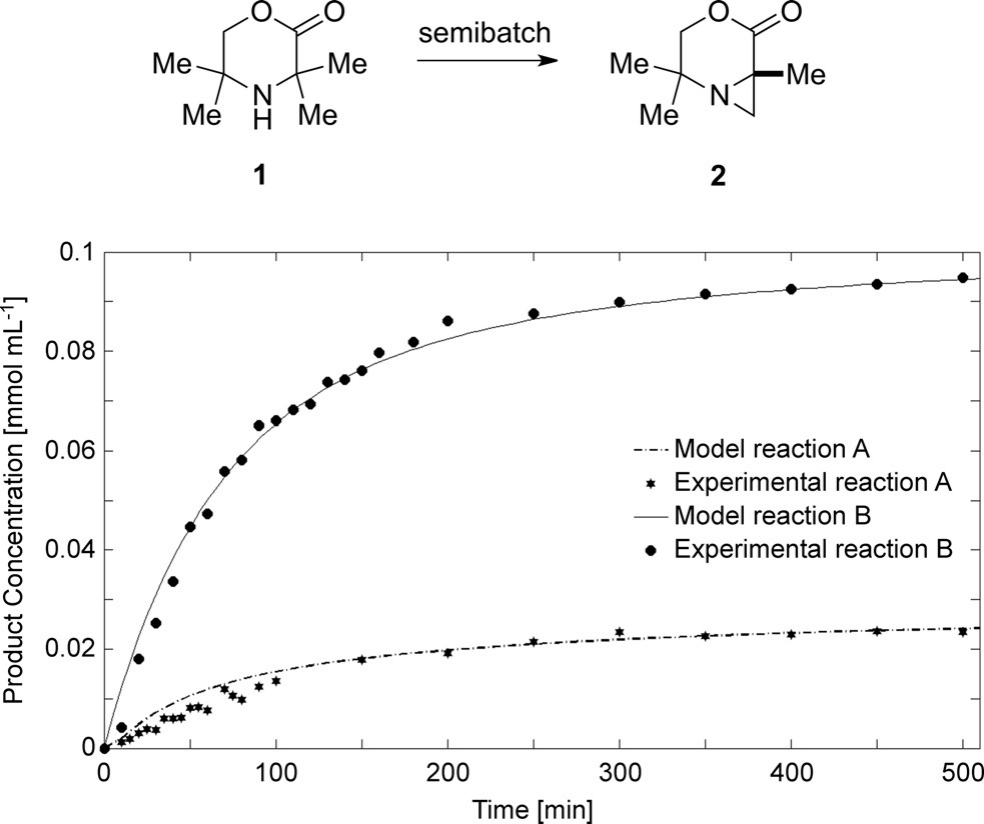
Comparison of the kinetic model with experimental results for two different semibatch reactions. Reaction conditions: *C*_Cat0_=0.005 mol L^−1^, *C*_Catfeed_=0 mol L^−1^, *F*=0.02 mL min^−1^, *T*=70 °C; Reaction A: *C*_SM0_=0.05 mol L^−1^; *C*_SMfeed_=0.1 mol L^−1^; Reaction B: *C*_SM0_=0 mol L^−1^, *C*_SMfeed_=0.025 mol L^−1^; SM: starting material; Cat: palladium acetate; 0: concentration in reactor before reaction start; feed: concentration in the feeding stream. Time “0” corresponds to when the pumping started.

The kinetic model was used to design a process model on the assumption that an ideal plug flow reactor was used. We defined two restrictive parameters: The reaction temperature could not be higher than 120 °C owing to the thermal instability of palladium acetate,^13^ and full conversion must be reached within 10 min for a large space–time yield to be attained. The model was used to select operating conditions that fulfilled both these requirements, while also considering that high acid concentrations may lead to degradation of the aziridine product (see Scheme 1 b).^7^ Optimization in silico by using the process model was performed with respect to the concentration of the starting material (amine **1**), Pd(OAc)_2_, and AcOH, and with respect to the temperature. The results led us to several possible sets of conditions, which were then tested experimentally. Our final process allowed a reduction of the catalyst loading to 0.5 mol % with a residence time of 10 min (Figure 3). We did not notice any by‐product formation over this time period.

**Figure 3:**
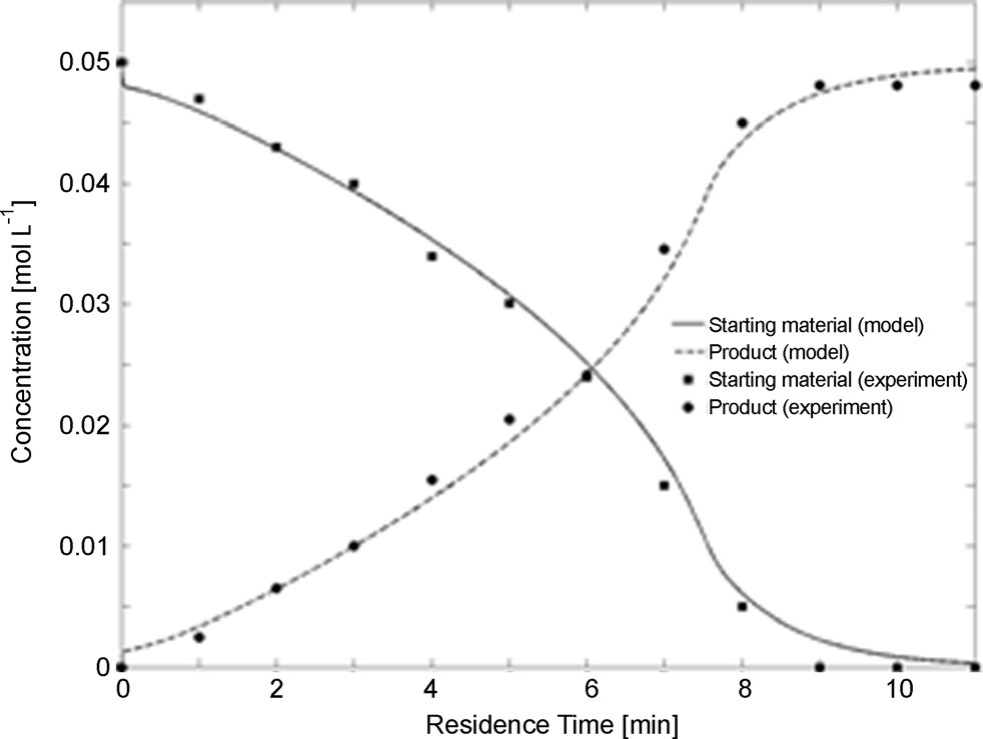
Results of continuous‐flow experiments under optimal conditions; *C*_SM_=0.05 mol L^−1^, *C*_Cat_=2.5 10^−4^ mol L^−1^, *C*_AcOH_=0.5 mol L^−1^. Concentrations were measured at the outlet of the reactor for different residence times in order to find the optimal residence time and compare experimental results with the process model.

Following optimization of the flow reaction, the design of a suitable process involving a purification method was undertaken. The aim was a gram‐scale synthesis characterized by a large space–time yield and the design of a convenient separation method for both the product and the catalyst (if possible, also suitable for catalyst recycling). The whole process should operate autonomously to be desirable for large‐scale applications. Since the catalytic system in batch is homogeneous, the same approach was initially applied to the flow process. We first tested the reaction on the microscale by using a temperature‐controlled custom‐made silicon‐glass microfluidic reactor. The use of a homogenous catalyst is beneficial in providing well‐defined active sites and, given effective mixing, rapid diffusivity to the catalyst. However, an efficient way of separating the catalyst from the products is required. Owing to the nature of the reaction system, a biphasic separation model was not possible. Furthermore, the most powerful, acid‐based, metal scavengers were not suitable for separating the Pd(OAc)_2_ from the reaction mixture because they would scavenge both the catalyst and the aziridine product. It was found that commercially available QuadraSil AP (with a pendant primary amine)^11^ successfully coordinated only to the palladium species, thus enabling removal of the catalyst from the spent reaction mixture. In a second separation step, the amine product could be separated from the reaction mixture by the use of a silica gel functionalized with strongly acidic groups. In this study we used the Isolute SCX‐3 gel (with pendant sulfonic acid groups)^12^ as a successful amine scavenger (Figure 4 a). The product can be washed on the gel and subsequently removed by the use of an eluent with a high pH value. Implementation of this “catch and release” technique yielded a product of high purity (Figure 4 b).

**Figure 4:**
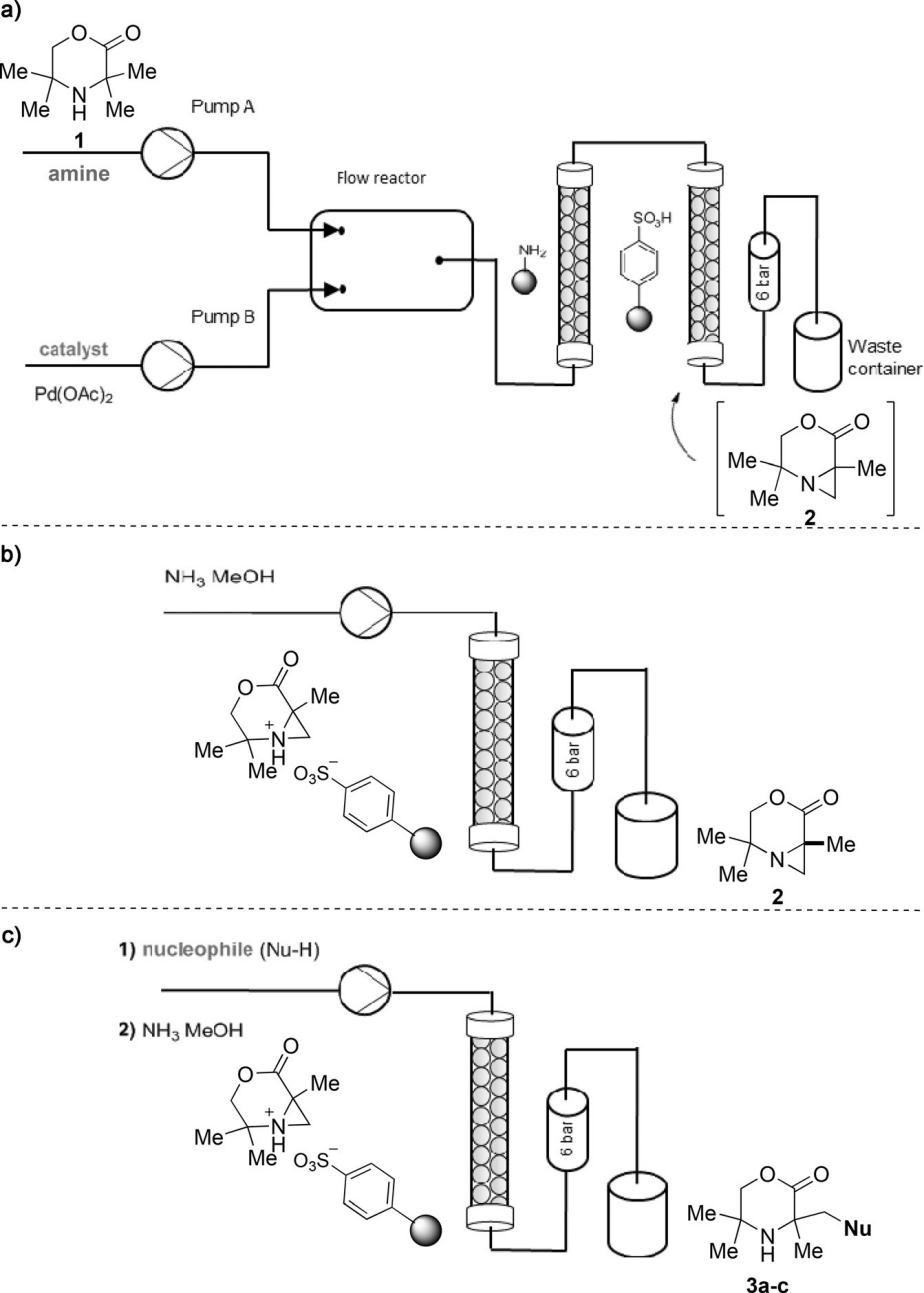
a) Flow process for the aziridination C−H activation reaction. Flow reactor: silicon‐glass microfluidic reactor or PTFE tubular reactor. The first column separates the catalyst from the spent mixture; the second column separates the product. Reactants were pumped by: pump A (starting material, oxidant), pump B (catalyst, acetic acid, acetic anhydride). A 6 bar pressure regulator was fitted. b) Elution of the product from the “catch” column. c) Aziridine ring opening and elution from the “catch” column to give functionalized morpholinones.

Following the successful development and optimization of a small‐scale process, we further tested the reaction on a larger scale by using a Vapourtec R‐Series system^14^ equipped with a 10 mL, thermostated poly(tetrafluoroethylene) (PTFE) tubular reactor. Full conversion was achieved with a flow rate equal to 1 mL min^−1^, which produced 4.68 mmol (771.9 mg) of aziridine product per hour, thus resulting in a space–time yield of 0.463 [kg L(reactor)^−1^ h^−1^]. The Vapourtec R‐Series is designed to operate autonomously, and the operational time is only limited by the capacity of the scavenger columns. This limitation can be easily overcome with a setup including two sets of columns: one operational and one in a stand‐by mode. To the best of our knowledge, no C−H activation of amines has been carried out previously in a flow system, and no C−H activation process has been performed on a multigram scale.^15^ Both experimental designs resulted in very good yields (isolation of products in 90 % yield), high purity of products (>90 % according to ^1^H NMR spectroscopy of the crude product against an internal standard), and, on the basis of inductively coupled plasma optical emission spectroscopy (ICP OES), concentrations of palladium in the mixture leaving the scavenger column below 1 ppm.

To extend the synthetic utility of this flow C−H activation procedure, we investigated the coupling of the C−H activation step to a subsequent reaction in a single flow process. It had been previously shown that the aziridine products could be functionalized through ring‐opening reactions with various nucleophiles, such as carboxylic acids, azides, thiols, and halogens.^6^, ^16^ To further highlight the benefits of translating this batch process into flow, we aimed to develop a robust procedure suitable for opening aziridines with weak nucleophiles and reagents that could be potentially hazardous when used in batch.

In our previous study, the ring‐opening reaction required the action of either a strong Brønsted or Lewis acid to activate the aziridine. In the current system, we questioned whether the strong sulfonic acid scavenging resin might also function as an activating agent for aziridine ring opening, thereby reducing the number of chemical operations and streamlining the overall transformation. The first step towards this goal was achieved by retaining the aziridine on the “catch” Isolute SCX‐3 column. While on the column, the aziridine is immobilized by sulfonic acid protonation, which makes it susceptible to nucleophilic attack: heating to just 60 °C is required to facilitate such a reaction. When a nucleophile—water, methanol, or hydrazoic acid generated in situ—was pumped through the resulting column (Figure 4 c), the derivatized products **3 a**–**c** were obtained in good yields (Figure 5). The use of a solid‐state support significantly facilitated the reaction procedure, minimized the number of purification steps, and shortened the reaction time considerably.

**Figure 5:**
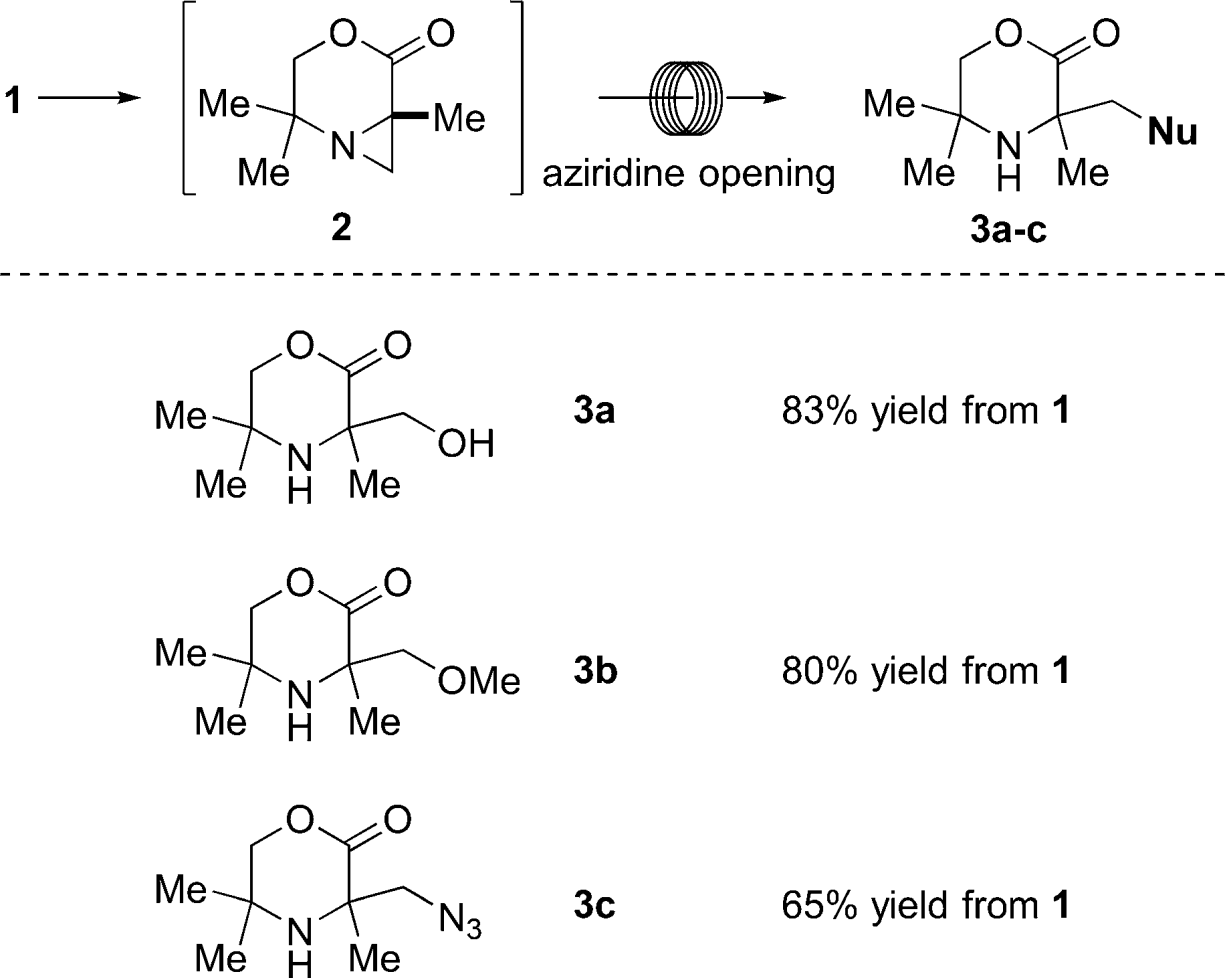
Scope of the aziridine‐ring‐opening reaction. Yields are given for the isolated product after the two‐step synthesis.

Although several reactions have been described previously for aziridine ring opening in continuous flow,^17^ to the best of our knowledge, none have been performed by using an immobilization–activation strategy. The high purity of the crude mixture (>90 %, ^1^H NMR) allows direct transformations of the synthesized amines without any additional treatment. This protocol could be used more generally for the opening of various aziridines through an effective “catch–release” flow process. Furthermore, otherwise time‐consuming and hazardous reactions with hydrazoic acid precursors can be conducted much more safely.

Following the successful application of homogeneous catalysis, we briefly investigated the use of a heterogeneous catalyst. The use of palladium(II) salts for the aziridination reaction significantly narrowed the choice of commercially available heterogeneous catalysts. However, satisfying results were observed with polymer‐bound dichlorobis(triphenylphosphine)palladium(II); ICP OES measurements of the spent reaction mixture showed palladium leaching below 5 mol %. There are very few reports of heterogeneous Pd^II^ catalysts, especially those that are resistant to leaching, which is a general limitation of this aspect of flow chemistry. Therefore, although this level of leaching cannot exclude the possibility that the reaction is catalyzed by solubilized species, the preliminary result suggests that heterogeneous catalysis is viable for the flow C−H activation process.

In summary, as a result of extensive kinetic and mechanistic investigations involving DFT studies, we have successfully performed a two‐step continuous‐flow synthesis of substituted morpholinones on the basis of C−H activation. By designing the process from first principles, we significantly shortened the overall reaction time and increased the space–time yield, while also reducing the number of purification steps. A thorough understanding of the reaction system allowed us to shorten the optimization time and efficiently design the flow process. Novel aspects of the process include the implementation of a C(sp^3^)−H activation reaction in a continuous‐flow system and its performance on a multigram scale. Our study further illustrates the utility of flow chemistry as a tool for functionalization through consecutive reactions. A further highlight is the nucleophilic opening of immobilized aziridines in flow.

## Supporting information

As a service to our authors and readers, this journal provides supporting information supplied by the authors. Such materials are peer reviewed and may be re‐organized for online delivery, but are not copy‐edited or typeset. Technical support issues arising from supporting information (other than missing files) should be addressed to the authors.

Supplementary

## References

[1a] C. Jiménez-González , P. Poechlauer , Q. B. Broxterman , B. S. Yang , D. am Ende , J. Baird , C. Bertsch , R. E. Hannah , P. Dell'Orco , H. Noorrnan , S. Yee , R. Reintjens , A. Wells , V. Massonneau , J. Manley , Org. Process Res. Dev. 2011, 15, 900–911; for a selection of reviews on flow chemistry, see:

[1b] R. L. Hartman , J. P. McMullen , K. F. Jensen , Angew. Chem. Int. Ed. 2011, 50, 7502–7519; 10.1002/anie.201004637 21710673

[1c] J. Wegner , S. Ceylan , A. Kirschning , Chem. Commun. 2011, 47, 4583–4592; 10.1039/c0cc05060a 21409184

[1d] J. Wegner , S. Ceylan , A. Kirschning , Adv. Synth. Catal. 2012, 354, 17–57;

[1e] K. S. Elvira , X. C. Solvas , R. C. R. Wootton , A. J. deMello , Nat. Chem. 2013, 5, 905–915; 24153367 10.1038/nchem.1753

[1f] J. C. Pastre , D. L. Browne , S. V. Ley , Chem. Soc. Rev. 2013, 42, 8849–8869; 23999700 10.1039/c3cs60246j

[1g] B. Gutmann , D. Cantillo , C. O. Kappe , Angew. Chem. Int. Ed. 2015, 54, 6688–6728; 10.1002/anie.201409318 25989203

[1h] T. Noël , S. L. Buchwald , Chem. Soc. Rev. 2011, 40, 5010–5029; 21826351 10.1039/c1cs15075h

[1i] D. Webb , T. F. Jamison , Chem. Sci. 2010, 1, 675–680;

[1j] S. Zelentsov , V. Hessel , E. Shahbazali , T. Noël , ChemBioEng Rev. 2014, 1, 230–240;

[1k] V. Hessel , E. Shahbazali , T. Noël , S. Zelentsov , ChemBioEng Rev. 2014, 1, 244–261.

[2] U. Hintermair , G. Francio , W. Leitner , Chem. Commun. 2011, 47, 3691–3701. 10.1039/c0cc04958a 21270995

[3] A. A. Lapkin , A. Voutchkova , P. Anastas , Chem. Eng. Process. 2011, 50, 1027–1034.

[4] For a selection of reviews on C−H activation, see:

[4a] A. D. Ryabov , Chem. Rev. 1990, 90, 403–424;

[4b] C. Jia , T. Kitamura , Y. Fujiwara , Acc. Chem. Res. 2001, 34, 633–639; 11513570 10.1021/ar000209h

[4c] K. Godula , D. Sames , Science 2006, 312, 67–72; 16601184 10.1126/science.1114731

[4d] H. M. L. Davies , J. R. Manning , Nature 2008, 451, 417–424; 18216847 10.1038/nature06485 PMC3033428

[4e] X. Chen , K. M. Engle , D. H. Wang , J. Q. Yu , Angew. Chem. Int. Ed. 2009, 48, 5094–5115; 10.1002/anie.200806273 PMC2722958 19557755

[4f] H. M. L. Davies , J. Du Bois , J. Q. Yu , Chem. Soc. Rev. 2011, 40, 1855–1856; 21390392 10.1039/c1cs90010b

[4g] J. Yamaguchi , A. D. Yamaguchi , K. Itami , Angew. Chem. Int. Ed. 2012, 51, 8960–9009; 10.1002/anie.201201666 22887739

[4h] L. McMurray , F. O'Hara , M. J. Gaunt , Chem. Soc. Rev. 2011, 40, 1885–1898. 21390391 10.1039/c1cs15013h

[5] D. R. Gauthier, Jr. , J. Limanto , P. N. Devine , R. A. Desmond , R. H. Szumigala, Jr. , B. S. Foster , R. P. Volante , J. Org. Chem. 2005, 70, 5938–5945; 16018689 10.1021/jo0507035

[6] A. McNally , B. Haffemayer , B. S. L. Collins , M. J. Gaunt , Nature 2014, 510, 129–133. 24870240 10.1038/nature13389

[7] A. P. Smalley , M. J. Gaunt , J. Am. Chem. Soc. 2015, 137, 10632–10641. 26247373 10.1021/jacs.5b05529

[8] E. G. Moschetta , S. Negretti , K. M. Chepiga , N. A. Brunelli , Y. Labreche , Y. Feng , F. Rezaei , P. Lively , H. M. L. Davies , C. W. Jones , Angew. Chem. Int. Ed. 2015, 54, 6470–6474; 10.1002/anie.201500841 25865826

[9] Gaussian 09 (Revision D.01), M. J. Frisch, et al., Gaussian, Inc., Wallingford CT, **2013**.

[10a] D. L. Davies , S. M. A. Donald , S. A. Macgregor , J. Am. Chem. Soc. 2005, 127, 13754–13755; 16201772 10.1021/ja052047w

[10b] M. Lafrance , C. N. Rowley , T. K. Woo , K. Fagnou , J. Am. Chem. Soc. 2006, 128, 8754–8756; 16819868 10.1021/ja062509l

[10c] D. García-Cuadrado , A. A. C. Braga , F. Maseras , A. M. Echavarren , J. Am. Chem. Soc. 2006, 128, 1066–1067; 16433509 10.1021/ja056165v

[10d] M. Lafrance , S. I. Gorelsky , K. Fagnou , J. Am. Chem. Soc. 2007, 129, 14570–14571; 17985911 10.1021/ja076588s

[10e] D. Balcells , E. Clot , O. Eisenstein , Chem. Rev. 2010, 110, 749–823; 20067255 10.1021/cr900315k

[10f] L. Ackermann , Chem. Rev. 2011, 111, 1315–1345. 21391562 10.1021/cr100412j

[11a] W. Solodenko , A. Doppiu , R. Frankfurter , C. Vogt , A. Kirschning , Aust. J. Chem. 2013, 66, 183–191;

[11b] A. Hinchcliffe , C. Hughes , D. A. Pears , M. R. Pitts , Org. Process Res. Dev. 2007, 11, 477–481;

[11c] http://Jmcct.com, “Silica-based spherical metal scavengers—Scavenging Technologies”, can be found under http://jmcct.com/products-services/product_p455.html, **2015**.

[12] http://Biotage.com, “Biotage—ISOLUTE SCX-3′, can be found under http://www.biotage.com/product-page/isolute-scx-3, **2015**.

[13] T. Tano , K. Esumi , K. Meguro , J. Colloid Interface Sci. 1989, 133, 530–533.

[14] http://Vapourtec.co.uk, “Vapourtec R-Series Flow Chemistry System | http://www.vapourtec.co.uk”, can be found under http://www.vapourtec.co.uk/products/rseriessystem, **2015**.

[15a] M. Christakakou , M. Schön , M. Schnürch , M. D. Mihovilovic , Synlett 2013, 24, 2411–2418;

[15b] L. Zhang , M. Geng , P. Teng , D. Zhao , X. Lu , J. Li , Ultrason. Sonochem. 2012, 19, 250–256; 21855388 10.1016/j.ultsonch.2011.07.008

[15c] H. P. L. Gemoets , V. Hessel , T. Noël , Org. Lett. 2014, 16, 5800–5803; 25341623 10.1021/ol502910e

[15d] N. Erdmann , Y. Su , B. Bosmans , V. Hessel , T. Noël , Org. Process Res. Dev. 2016, 20, 831–835.

[16] X. E. Hu , Tetrahedron 2004, 60, 2701–2743.

[17] N. Hsueh , G. J. Clarkson , M. Shipman , Org. Lett. 2015, 17, 3632–3635. 26158313 10.1021/acs.orglett.5b01777

