# Supplementary material for: Continuous‐Flow Synthesis and Derivatization of Aziridines through Palladium‐Catalyzed C(sp3)−H Activation
Source: Angew Chem Int Ed Engl. 2016 Jun 15;55(31):8878–83. doi: 10.1002/anie.201602483 (PMC5094502; doi:10.1002/anie.201602483)
Supplement: Supplementary file 1 — Supplementary [file ANIE-55-8878-s001.pdf]

Angewandte Chemie
Eine Zeitschrift der Gesellschaft Deutscher Chemiker

Supporting Information

Continuous-Flow Synthesis and Derivatization of Aziridines through Palladium-Catalyzed C(sp^3)–H Activation

Jacek Zakrzewski, Adam P. Smalley, Mikhail A. Kabeshov, Matthew J. Gaunt, and Alexei A. Lapkin**

anie_201602483_sm_miscellaneous_information.pdf

1. General Information

Proton nuclear magnetic resonance (^{1}H NMR) spectra were recorded at ambient temperature on a Bruker AM 400 (400 MHz) or an Avance 500 (500 MHz) spectrometer. Chemical shifts (δ) are reported in ppm and quoted to the nearest 0.01 ppm relative to the residual protons in $CDCl_3$ (7.24 ppm) and coupling constants (J) are quoted in Hertz (Hz). Data are reported as follows: Chemical shift (multiplicity, coupling constants, number of protons). Coupling constants were quoted to the nearest 0.1 Hz and multiplicity reported according to the following convention: s = singlet, d = doublet, t = triplet, q = quartet, qn = quintet, sp = septet, m = multiplet, br = broad; and associated combinations, e.g. dd = doublet of doublets. Where coincident coupling constants have been observed, the apparent (app) multiplicity of the proton resonance has been reported.

Carbon nuclear magnetic resonance (^{13}C NMR) spectra were recorded at ambient temperature on a Bruker AM 400 (101 MHz) or an Avance 500 (126 MHz) spectrometer. Chemical shift (δ) was measured in ppm and quoted to the nearest 0.1 ppm relative to the residual solvent peaks in $CDCl_3$ (77.16 ppm).

High resolution mass spectra (HRMS) were measured at the EPSRC Mass Spectrometry Service at the University of Swansea.

Infared (IR) spectra were recorded on a Perkin Elmer 1FT-IR Spectrometer fitted with an ATR sampling accessory as either solids or neat films, either through direct application or deposited in $CHCl_3$, with absorptions reported in wavenumbers (cm^{-1}).

Analytical thin layer chromatography (TLC) was performed using pre-coated Merck glass backed silica gel plates (Silicagel 60 F254). Flash column chromatography was undertaken on Fluka or Material Harvest silica gel (230–400 mesh) under a positive pressure of nitrogen unless otherwise stated. Basic alumina was obtained from Merck as aluminium oxide 90 standardized. Visualization was achieved using chemical staining with basic potassium permanganate solutions. All reagents were purchased at the highest commercial quality and used without further purification. Reactions were carried out under an inert atmosphere of N_2 unless otherwise stated. All reactions were monitored by TLC, ^{1}H NMR spectra taken from reaction samples and gas chromatography (GC) using an Agilent 6850 gas chromatograph for FID analysis.

2. Substrate Synthesis

3,3,5,5-Tetramethylmorpholin-2-one **1**

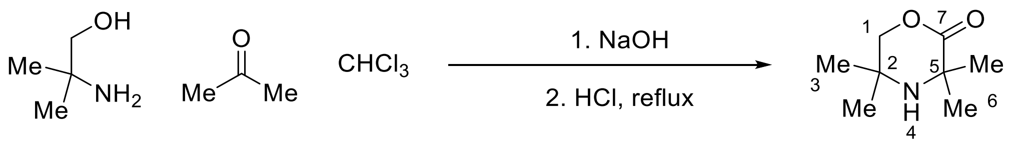

2-Amino-2-methyl-1-propanol (10.0 g, 112 mmol, 1.0 equiv), acetone (82 ml, 1.12 mol, 10.0 equiv) and chloroform (13.5 ml, 168 mmol, 1.5 equiv) were added under nitrogen to a 500 ml three neck flask equipped with a thermometer. The mixture was cooled with an acetone/ice bath and stirred vigorously. Powdered sodium hydroxide (22.4 g, 560 mmol, 5.0 equiv) was then added portion-wise while keeping the internal temperature below 5 °C. After completion of the addition, the thick solution was vigorously stirred for 2 hours, keeping the internal temperature below 10 °C. The solution was then allowed to warm to room temperature over 16 hours. The white solid was filtered, rinsed with acetone and twice in methanol (2 x 100 ml). The combined filtrates were concentrated under vacuum to afford the crude sodium carboxylate which was then refluxed for 6 hours in concentrated hydrochloric acid (100 ml). After cooling to room temperature, the hydrochloric acid was removed *in vacuo*. The flask was then placed in an ice bath and a saturated solution of sodium bicarbonate was carefully added until the mixture became basic. The solution was extracted with ethyl acetate (3 x 50 ml) and the combined organics were washed with brine (50 ml), dried ($MgSO_4$) and concentrated *in vacuo*. The product was purified by Kugelrohr distillation (0.38 mbar, ~95 °C) to give a colourless liquid (8.9 g, 56.7 mmol, 50.6%).

^{1}H NMR (400 MHz, $CDCl_3$): δ 4.10 (s, 2H, H-1), 1.36 (s, 6H, H-6), 1.12 (s, 6H, H-3); ^{13}C NMR (100 MHz, $CDCl_3$): δ 175.2 (C-7), 78.1 (C-1), 54.6 (C-5), 49.1 (C-2), 30.6 (C-6), 26.4 (C-3); IR ν_{max}/cm^{-1} (film): 3336 (N–H), 2973, 1726 (C=O), 1473, 1399, 1379, 1286, 1259, 1235, 1196, 1127, 1047, 915, 890, 806, 751

Characterization consistent with previous literature.[1]

3. Aziridination Procedures

2,2,6-Trimethyl-4-oxa-1-azabicyclo[4.1.0]heptan-5-one **2**

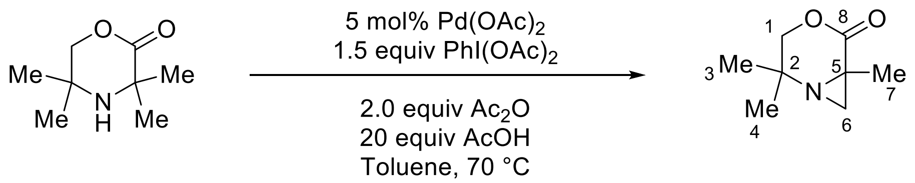

Batch reactions:

A microwave vial equipped with a stir bar was charged with $Pd(OAc)_2$ (5.6 mg, 0.025 mmol, 0.05 equiv) and $PhI(OAc)_2$ (241.6 mg, 0.75 mmol, 1.5 equiv). Toluene (4.3 ml, 0.1 M) was added followed by acetic acid (570 µl, 10 mmol, 20 equiv) and acetic anhydride (95 µl, 0.50 mmol, 2.0 equiv) and the vial was sealed with a Teflon cap. The vessel was placed in a pre-heated oil bath at 70 °C for 30 min and stirred. Afterwards, 3,3,5,5- tetramethylmorpholin-2-one **1** (78.6 mg, 0.5 mmol, 1.0 equiv) was added and the reaction mixture stirred for a further 150 min. The solution was filtered through Celite, eluting with ethyl acetate (~50 ml), and concentrated *in vacuo*. The product was purified by column chromatography (Al_2O_3, 10%-20% EtOAc:hexane, R_f = 0.13 (SiO_2, 10% Et_2O:CH_2Cl_2)) to give the product as colourless crystals (63.6 mg, 0.41 mmol, 82%).

^{1}H NMR (400 MHz, $CDCl_3$): δ 3.99 (d, J = 12.1 Hz, 1H, H-1), 3.87 (d, J = 12.1 Hz, 1H, H-1), 2.45 (s, 1H, H-6), 1.90 (s, 1H, H-6), 1.45 (s, 3H, H-7), 1.29 (s, 3H, H-3/4), 1.13 (s, 3H, H-3/4); ^{13}C NMR (100 MHz, $CDCl_3$): δ 170.8 (C-8), 71.7 (C-1), 50.0 (C- 5), 36.0 (C-2), 32.9 (C-6), 25.6 (C-7), 23.7 (C-3/4), 20.9 (C-3/4); IR ν_{max}/cm^{-1} (film): 2973, 2934, 1721 (C=O), 1498, 1467, 1406, 1378, 1327, 1303, 1283, 1255, 1217, 1198, 1131, 1056, 1041, 981, 956, 905, 848, 768, 745, 700

Characterization consistent with previous literature.[1]

Flow experiments:

Stream one (toluene (10 ml), acetic acid (0.7 ml, 10 mmol), acetic anhydride (200 µl, 0.50 mmol), 3,3,5,5- tetramethylmorpholin-2-one **1** (314.4 mg, 2 mmol), $PhI(OAc)_2$ (966.4 mg, 3 mmol)) was pumped using a Knauer HPLC Pump; stream two (toluene (10 ml), acetic acid (0.7 ml, 10 mmol), acetic anhydride (200 µl, 0.50 mmol), palladium acetate (2.2 mg, 0.01 mmol)) was pumped using a second Knauer HPLC pump. Streams were mixed using a static Y-shaped mixer and directed into a thermostated flow reactor at 120 ^{o}C (a 6 bar BPR was fitted). The flow rates (equal for both streams) were adjusted accordingly in order to achieve 10 min residence time. The spent mixture was directed into an Omnifit column kept at room temperature and packed using Quadrasil AP gel (in order to separate the catalyst); afterwards the spent mixture was directed into a second Omnifit column kept at room temperature and packed with Isolute SCX-3 gel (in order to catch the product). The spent mixture was then directed into a waste container. When the reaction was finished, the system was flushed with toluene (30 ml) and methanol (50 ml). Following that, the second Omnifit column was flushed with 20 ml of NH_3 7N in MeOH in order to elute the product. The solution was concentrated *in vacuo* and purified by column chromatography (Al_2O_3, 10%-20% EtOAc:hexane, R_f = 0.13 (SiO_2, 10% Et_2O:CH_2Cl_2)) to give the product as colourless crystals (278.9 mg, 1.79 mmol, 90%).

Characterization consistent with batch experiments.

Up to our knowledge, no additional safety precautions must be undertaken while handling synthesised aziridines (even at high temperatures). However, since they were not properly tested and they do not have COSHH data, we treated them as toxic with standard PPE used at any time.

4. Nucleophilic ring opening

4.1 3-(Hydroxymethyl)-3,5,5-trimethylmorpholin-2-one **3a**

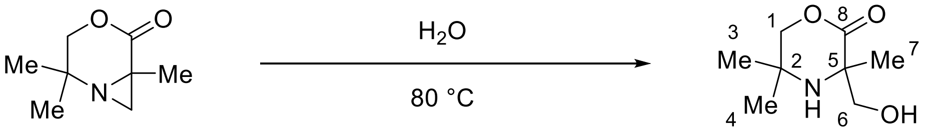

2,2,6-Trimethyl-4-oxa-1-azabicyclo[4.1.0]heptan-5-one **2** (100 mg, 0.65 mmol) was dissolved in toluene (10 ml) and the solution was pumped into an Omnifit column packed with Isolute SCX-3 gel.* The column was flushed with methanol (20 ml), deionized water (50 ml) and heated using Vapourec R-Series to 80 °C. After reaching the set temperature, deionized water was pumped through the column at a flowrate equal to 0.1 [ml/min]. After 60 min the column was cooled down to room temperature and flushed with MeOH (10 ml). The products were eluted from the column using NH_3 7N in MeOH (20 ml) and concentrated *in vacuo*. Purification by column chromatography was performed (SiO_2, 50% Et_2O:CH_2Cl_2, R_f = 0.15) to give the product as a colourless oil (108.0 mg, 0.61 mmol, 94%).

*The aziridine synthesised using the flow process, once caught on the initial Isolute SCX-3 gel Omnifit column, could be used directly in this procedure (i.e without needing to be eluted in the previous step and subsequently loaded onto a fresh column).

^{1}H NMR (400 MHz, $CDCl_3$) δ: 4.14 (d, J =3.0 Hz, 1H, H-1), 4.13 (d, J =3.0 Hz, 1H, H-1), 3.70 (d, J = 3.0 Hz, 1H, H-6), 3.35 (d, J = 3.0 Hz, 1H, H-6), 1.38 (s, 3H, H-7), 1.24 (s, 3H, H-3/4), 1.17 (s, 3H, H3/4); ^{13}C NMR (100 MHz, $CDCl_3$) δ: 173.9, 77.6, 69.2, 58.9, 48.9, 26.2, 26.1, 25.6; IR v_{max}/cm^{-1}(film): 3313 (br, O–H), 2974, 1721 (C=O), 1284, 1047; m/z HRMS (ESI) found $[M+H]^+$ 174.1121, $C_8H_{16}NO_3$ requires 174.1125

4.2 3-(Methoxymethyl)-3,5,5-trimethylmorpholin-2-one **3b**

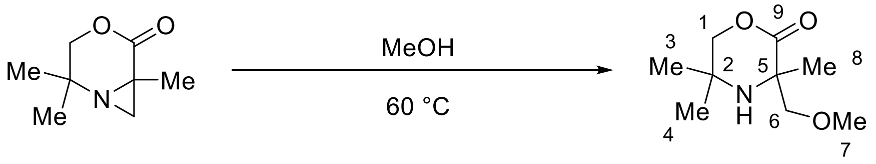

2,2,6-Trimethyl-4-oxa-1-azabicyclo[4.1.0]heptan-5-one **2** (100mg, 0.65 mmol) was dissolved in toluene (10 ml) and the solution was pumped into an Omnifit column packed with Isolute SCX-3 gel.* The column was flushed with dry methanol (20 ml, dried with Na_2SO_4) and heated using Vapourec R-Series to 60 °C. After reaching the set temperature, dry methanol was pumped through the column at a flowrate equal to 0.1 [ml/min]. After 60 min the column was cooled down to room temperature and the products were eluted from the column using NH_3 7N in MeOH (20 ml) and concentrated *in vacuo*. Purification by column chromatography was performed (SiO_2, 10% Et_2O:CH_2Cl_2, R_f = 0.22) to give the product as a colourless oil (101.2 mg, 0.58 mmol, 90%).

*The aziridine synthesised using the flow process, once caught on the initial Isolute SCX-3 gel Omnifit column, could be used directly in this procedure (i.e without needing to be eluted in the previous step and subsequently loaded onto a fresh column).

^{1}H NMR (400 MHz, $CDCl_3$) δ: 4.14 (d, J =2.0 Hz, 1H, H-1), 4.12 (d, J =2.0 Hz, 1H, H-1), 3.62 (d, J = 3.0 Hz, 1H, H-6), 3.33 (s, 3H, H-7), 3.21 (d, J = 3.0 Hz, 1H, H-6), 1.33 (s, 3H, H-8), 1.23 (s, 3H, H-3/4), 1.13 (s, 3H, H-3/4); ^{13}C NMR (100 MHz, $CDCl_3$) δ: 173.3, 79.6, 78.5; 59.2, 58.7, 48.5, 26.6, 26.1, 25.1; IR ν_{max}/cm^{-1}(film): 2970, 1733 (C=O), 1284, 1104, 1049; m/z HRMS (ESI) found $[M+H]^+$ 188.1279, $C_8H_{16}NO_3$ requires 188.1281

4.3 3-(Azidomethyl)-3,5,5-trimethylmorpholin-2-one **3c**

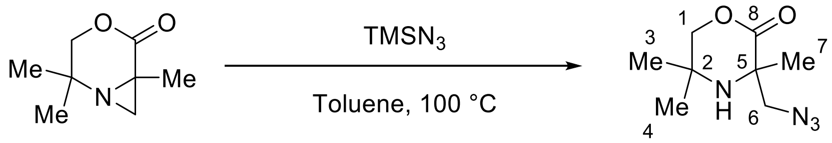

2,2,6-Trimethyl-4-oxa-1-azabicyclo[4.1.0]heptan-5-one **2** (100 mg, 0.65 mmol) was dissolved in toluene (10 ml) and the solution was pumped into an Omnifit column packed with Isolute SCX-3 gel.* The column was flushed with toluene (20 ml) and heated using Vapourec R-Series to 100 °C. After reaching the set temperature, the mixture of toluene and $TMSN_3$ was pumped through the column at a flowrate equal to 0.1 [ml/min] (30 ml of 50% $TMSN_3$ in toluene was recirculated over this period). After 5 h the column was cooled down to room temperature and flushed with MeOH (10 ml). The product was eluted from the column using NH_3 7N in MeOH (20 ml) and concentrated *in vacuo*. Purification by column chromatography was performed (SiO_2, 1% Et_2O:CH_2Cl_2, R_f = 0.48) to give the product as a colourless oil (95.5 mg, 0.48 mmol, 74%).

*The aziridine synthesised using the flow process, once caught on the initial Isolute SCX-3 gel Omnifit column, could be used directly in this procedure (i.e without needing to be eluted in the previous step and subsequently loaded onto a fresh column).

^{1}H NMR (400 MHz, $CDCl_3$) δ: 4.20 (d, J =2.0 Hz, 1H, H-1), 4.13 (d, J =2.0 Hz, 1H, H-1), 3.57 (d, J = 3.0 Hz, 1H, H-6), 3.20 (d, J = 3.0 Hz, 1H, H-6), 1.41 (s, 3H, H-7), 1.23 (s, 3H, H-3/4), 1.19 (s, 3H, H-3/4); ^{13}C NMR (100 MHz, $CDCl_3$) δ: 172.4, 78.0, 60.6, 58.8, 48.9, 27.2, 26.2, 26.0; IR ν_{max}/cm^{-1}(film): 3332, 2973, 2097, 1728, 1286, 1045; m/z HRMS: (ESI) found $[M+H]^+$ 199.1186, $C_8H_{15}N_4O$ requires 199.1190

5. Kinetic studies

Firstly, to ensure that the experiments were performed in a strictly kinetically controlled regime, three experiments, with agitation rate as the variable, were performed. The following conditions were used: C_{SM0}=0.05 mol l^{-1}; C_{Cat0}=0.005 mol l^{-1}; C_{AcOH}=0.7 mol l^{-1}. SM – starting material; Cat – palladium acetate; AcOH – acetic acid. Mixtures were prepared using the same stock solution and three different agitation rates were used: 300 RPM, 600 RPM and 900 RPM (Figure 1).

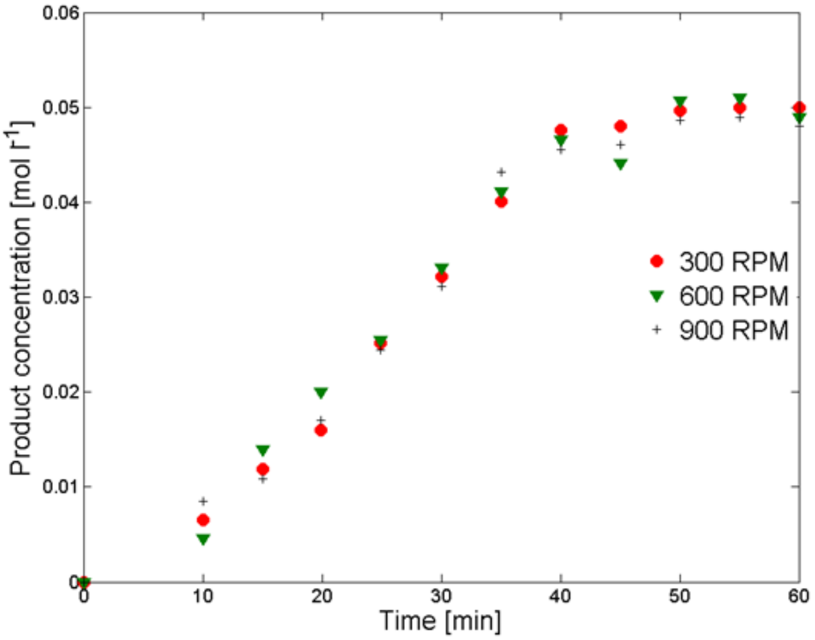

Figure 1. The influence of agitation on the reaction rate.

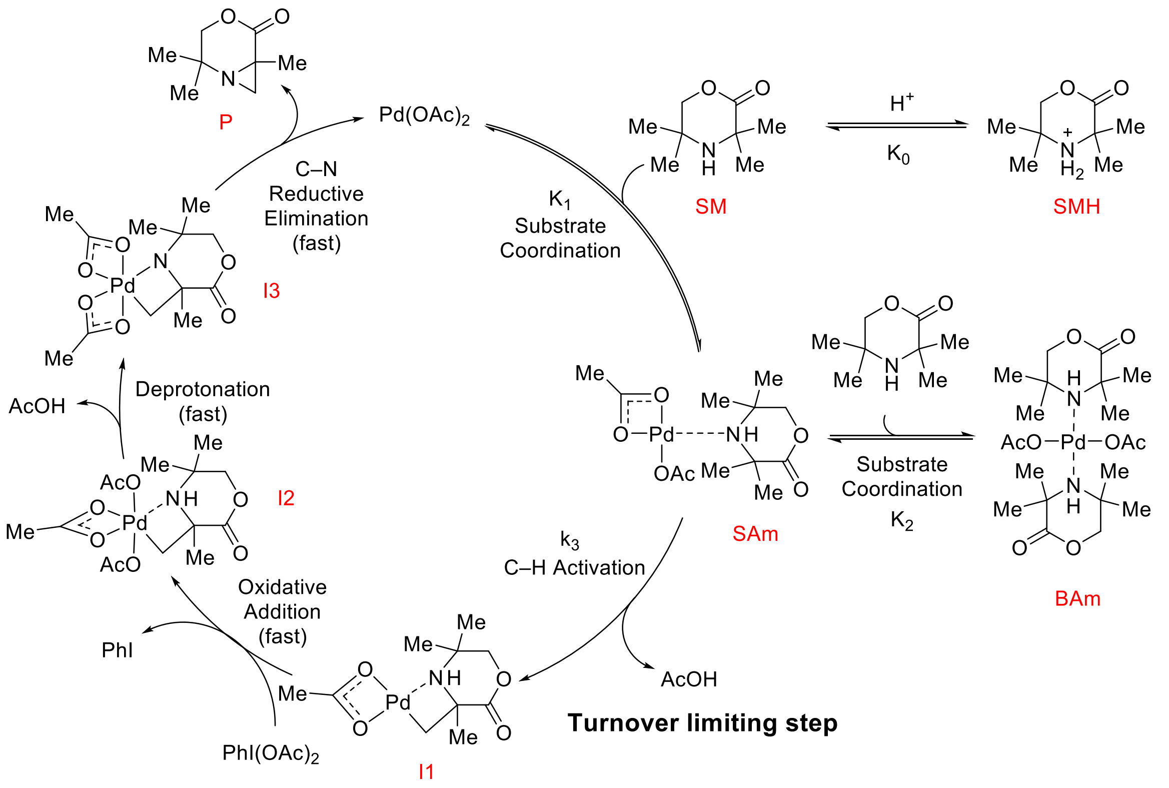

Figure 2 Mechanistic model of investigated reaction.

Following that, the mechanistic model (Figure 2) was reduced to the microkinetic model consisting of 10 ordinary differential equations:

$$\frac{dC_{Pd(OAc)_2}}{dt} = k_6 C_{I3} - k_1 C_{Pd(OAc)_2} C_{SM} + k_{-1} C_{Sam}$$

$$\frac{dC_{Sm}}{dt} = -k_0 C_{SM} C_{AcOH} + k_{-0} C_{SMH} - k_1 C_{Pd(OAc)_2} C_{SM} + k_{-1} C_{Sam}$$

$$-k_2 C_{Sam} C_{SM} + k_{-2} C_{Bam}$$

$$\frac{dC_{SMH}}{dt} = k_0 C_{SM} C_{AcOH} - k_{-0} C_{SMH}$$

$$\frac{dC_{Sam}}{dt} = k_1 C_{Pd(OAc)_2} C_{SM} - k_{-1} C_{Sam} - k_2 C_{Sam} C_{SM} + k_{-2} C_{Bam} - k_3 C_{Sam}$$

$$\frac{dC_{Bam}}{dt} = k_2 C_{Sam} C_{SM} - k_{-2} C_{Bam}$$

$$\frac{dC_{I1}}{dt} = k_3 C_{Sam} - k_4 C_{I1}$$

$$\frac{dC_{I2}}{dt} = k_4 C_{I1} - k_5 C_{I2}$$

$$\frac{dC_{I3}}{dt} = k_5 C_{I2} - k_6 C_{I3}$$

$$\frac{dC_P}{dt} = k_6 C_{I3}$$

$$\frac{dC_{AcOH}}{dt} = -k_0 C_{SM} C_{AcOH} + k_{-0} C_{SMH} + k_3 C_{Sam} + k_5 C_{I2}$$

and three equations:

$$K_0 = \frac{k_0}{k_{-0}}$$

$$K_1 = \frac{k_1}{k_{-1}}$$

$$K_2 = \frac{k_2}{k_{-2}}$$

Where:

$C_{Pd(OAc)2}$ is a concentration of free palladium acetate

C_{SM} is a concentration of starting material

C_{SMH} is a concentration of a salt of starting material and acetic acid

C_{Sam} is a concentration of a *single*-amine intermediate

C_{Bam} is a concentration of a *bis*-amine intermediate

C_{I1} is a concentration of intermediate 1 (after reaction k3)

C_{I2} is a concentration of intermediate 2 (after reaction k4)

C_{I3} is a concentration of intermediate 3 (after reaction k5)

C_P is a concentration of a product

C_{AcOH} is a concentration of acetic acid

Only parameters for reactions preceding the turnover limiting step were estimated. The rest were set as higher than k_3.

The following reactions were performed in order to build a database for kinetic parameter estimation:

No.	Palladium acetate [mol l^{-1}]	Substrate [mol l^{-1}]	Acetic acid [mol l^{-1}]	Temperature [^oC]
**0**	0.01	0.1	0	70
**1**	0.0025	0.05	0.7	70
**2**	0.0025	0.05	1.4	70
**3**	0.0025	0.05	2.1	70
**4**	0.0025	0.05	2.8	70
**5**	0.0025	0.05	3.5	70
**6**	0.005	0.05	0.7	70
**7**	0.005	0.05	1.4	70
**8**	0.005	0.05	2.1	70
**9**	0.005	0.05	2.8	70
**10**	0.005	0.05	3.5	70
**11**	0.00125	0.05	0.7	70
**12**	0.00125	0.05	1.4	70
**13**	0.00125	0.05	2.1	70
**14**	0.00125	0.05	2.8	70
**15**	0.00125	0.05	3.5	70
**16**	0.005	0.1	0.7	70
**17**	0.005	0.1	1.4	70
**18**	0.005	0.1	2.1	70
**19**	0.005	0.1	2.8	70
**20**	0.005	0.1	3.5	70
**21**	0.025	0.1	0.7	70
**22**	0.0025	0.1	1.4	70
**23**	0.0025	0.1	2.1	70
**24**	0.0025	0.1	2.8	70
**25**	0.0025	0.1	3.5	70
**26**	0.00125	0.1	0.7	70
**27**	0.00125	0.1	1.4	70
**28**	0.00125	0.1	2.1	70
**29**	0.00125	0.1	2.8	70
**30**	0.00125	0.1	3.5	70
**31**	0.005	0.2	0.7	70
**32**	0.005	0.2	1.4	70
**33**	0.005	0.2	2.1	70
**34**	0.005	0.2	2.8	70
**35**	0.005	0.2	3.5	70
**36**	0.005	0.05	0.7	80
**37**	0.005	0.05	0.7	90
**38**	0.005	0.05	0.7	100

Parameters were fitted against experimental data using gPROMS® Model Builder 4.0.0.

	Gibbs Free Energies [kcal/mol]	Kinetic parameters[a]
K_0	-0.78	3.14 [l mol-1min-1]
K_1	-10.16	2963160.29 [l mol-1min-1]
K_2	-6.07	7415.93 [l mol-1min-1]
k_3	18.55	9.23 [min-1]

Table 1. Results of parameter estimation. a) Values for 70°C.

Parameter	K_0	K_1	K_2	k_3
K_0	1			
K_1	0.241	1		
K_2	0.859	0.149	1	
k_3	-0.375	0.312	0.134	1

Table 2. Correlation matrix.

6. Fit between experimental data and model.

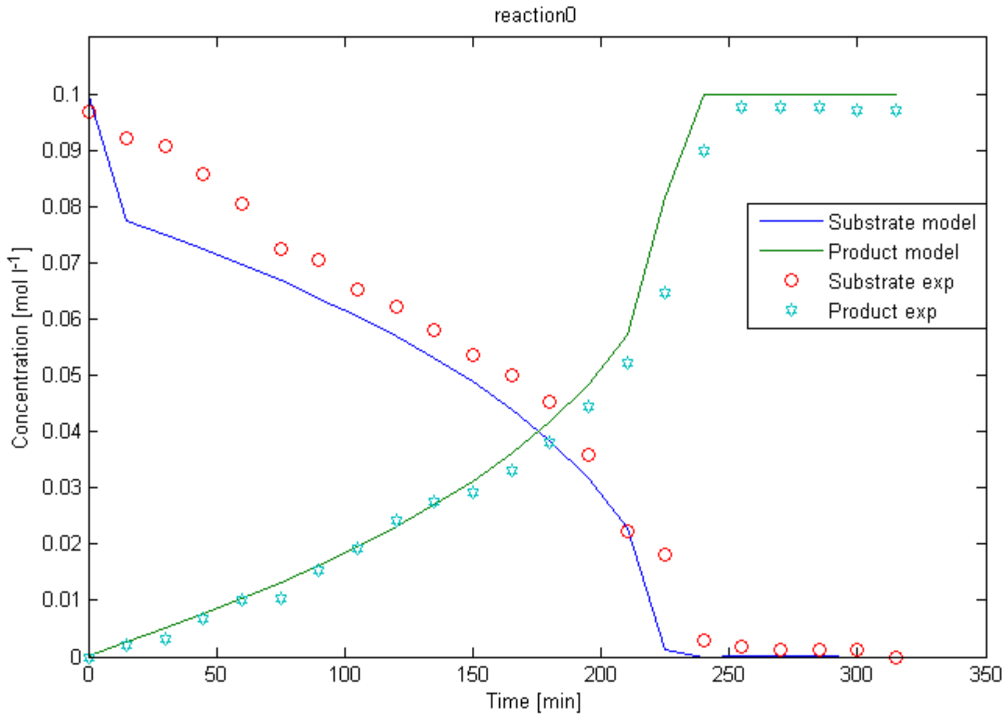

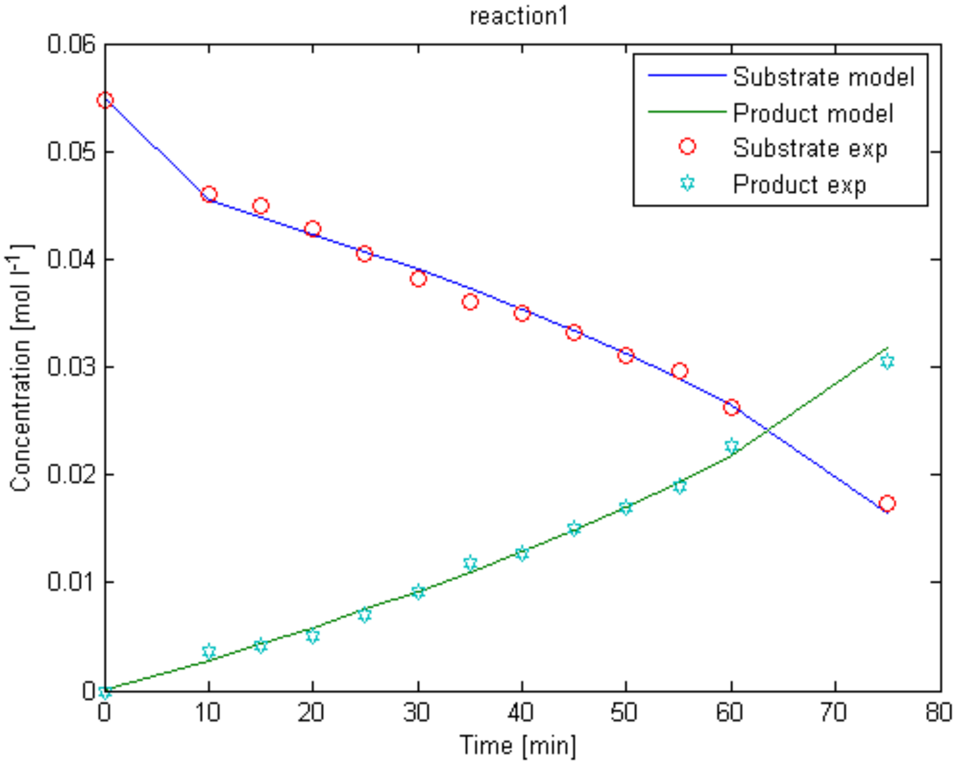

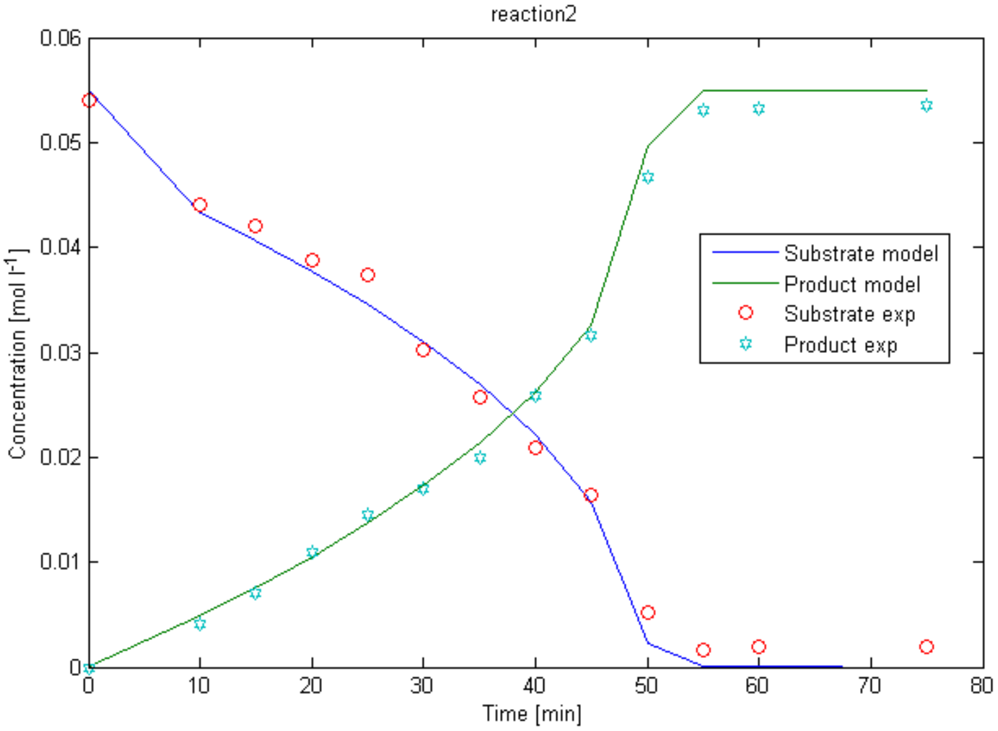
reaction2
Concentration [mol l-1]
Time [min]
Substrate model
Product model
Substrate exp
Product exp

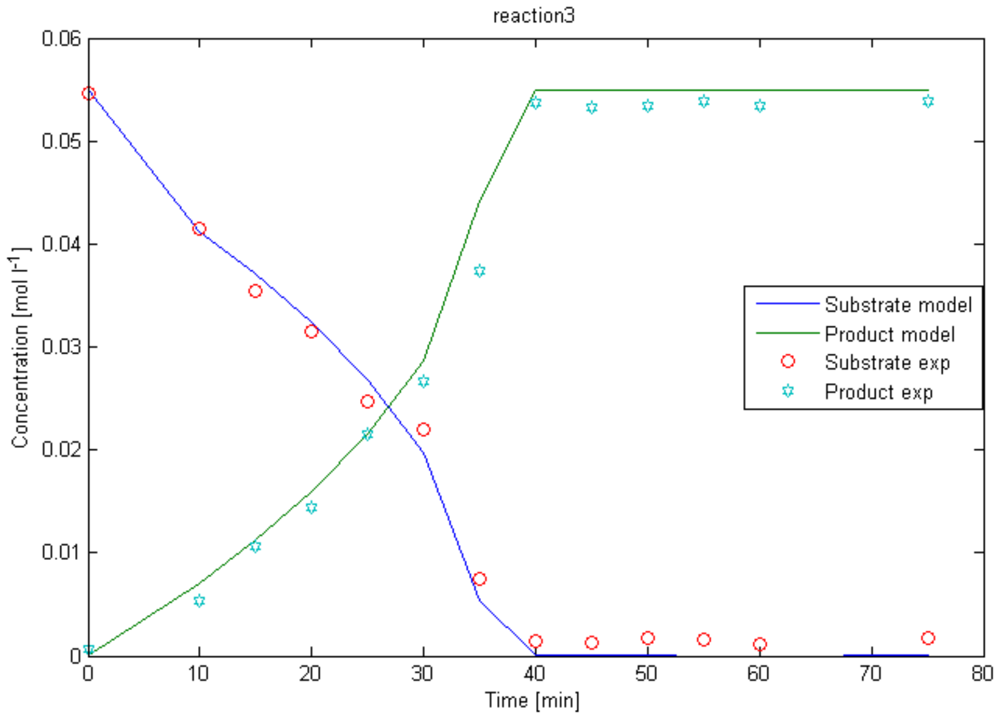
reaction3
Concentration [mol l-1]
Time [min]
Substrate model
Product model
Substrate exp
Product exp

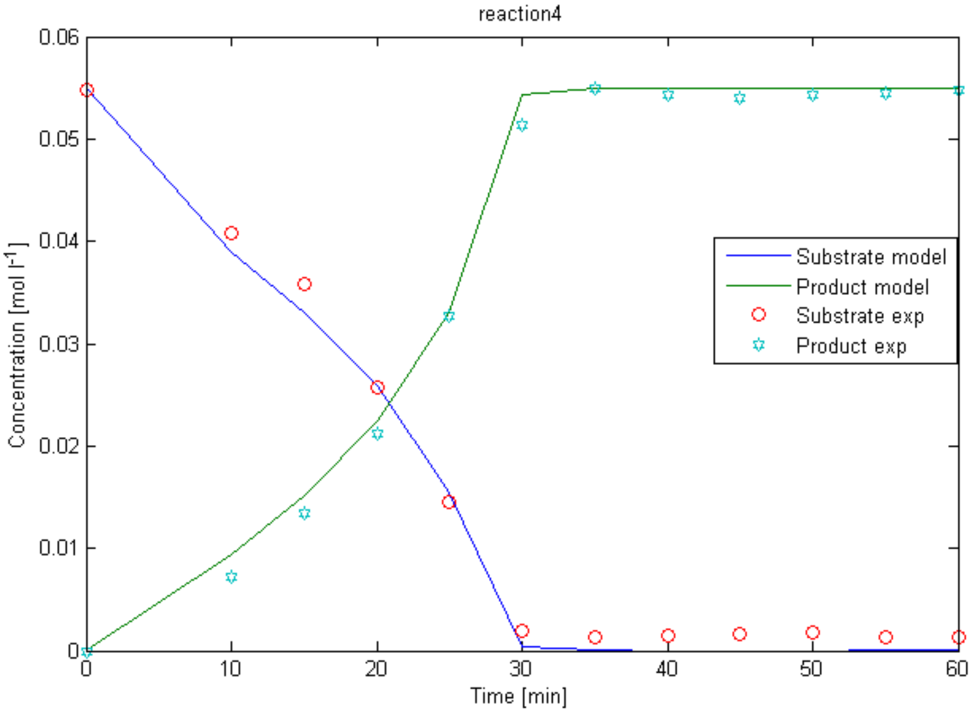
reaction4
Substrate model
Product model
Substrate exp
Product exp
Concentration [mol l-1]
Time [min]

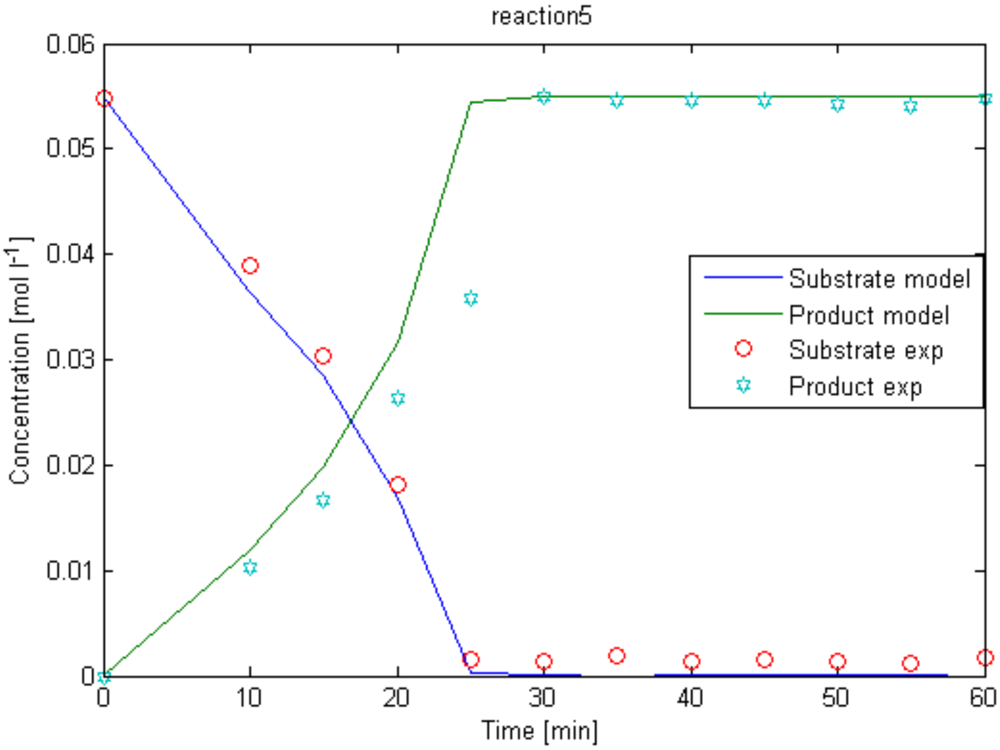
reaction5
Substrate model
Product model
Substrate exp
Product exp
Concentration [mol l-1]
Time [min]

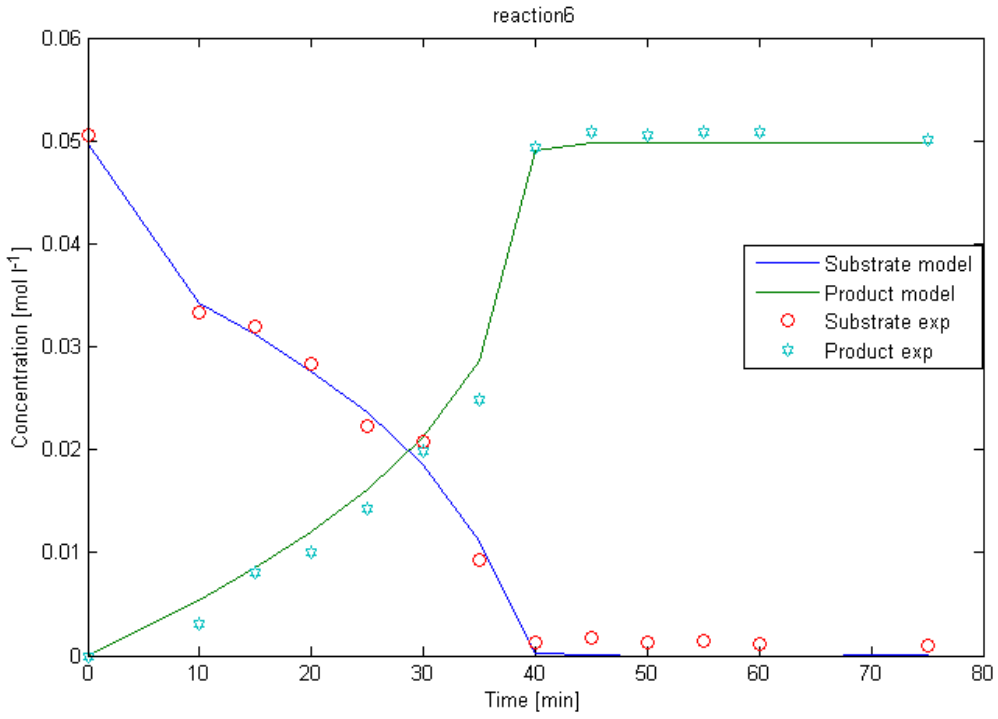
reaction6
0.06
0.05
0.04
0.03
0.02
0.01
0
Concentration [mol l-1]
0
10
20
30
40
50
60
70
80
Time [min]
Substrate model
Product model
Substrate exp
Product exp

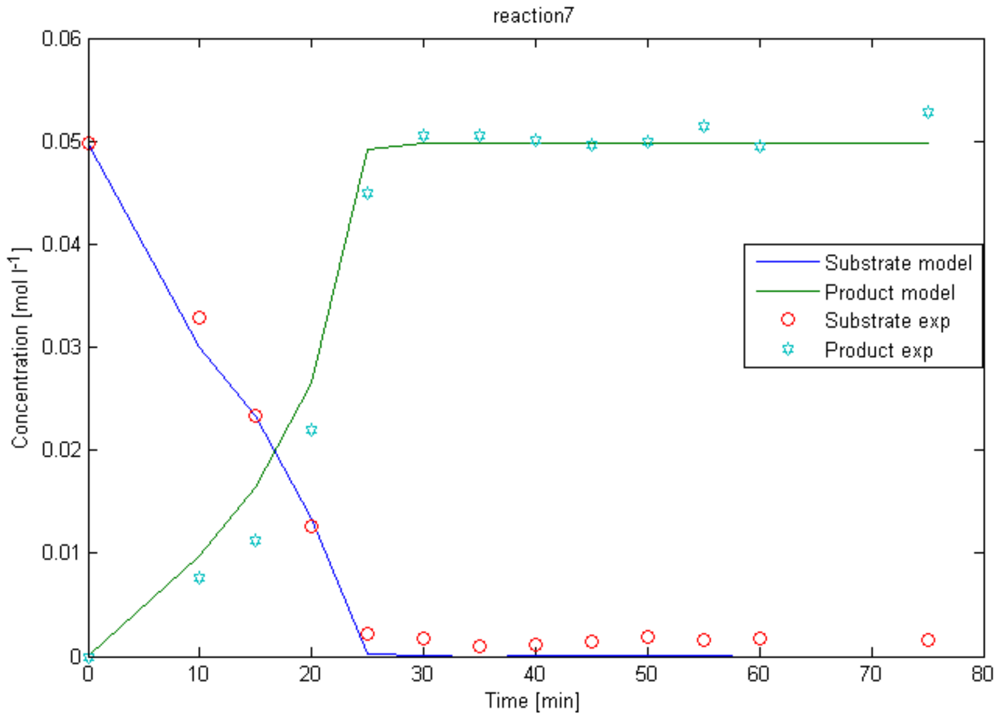
reaction7
0.06
0.05
0.04
0.03
0.02
0.01
0
Concentration [mol l-1]
0
10
20
30
40
50
60
70
80
Time [min]
Substrate model
Product model
Substrate exp
Product exp

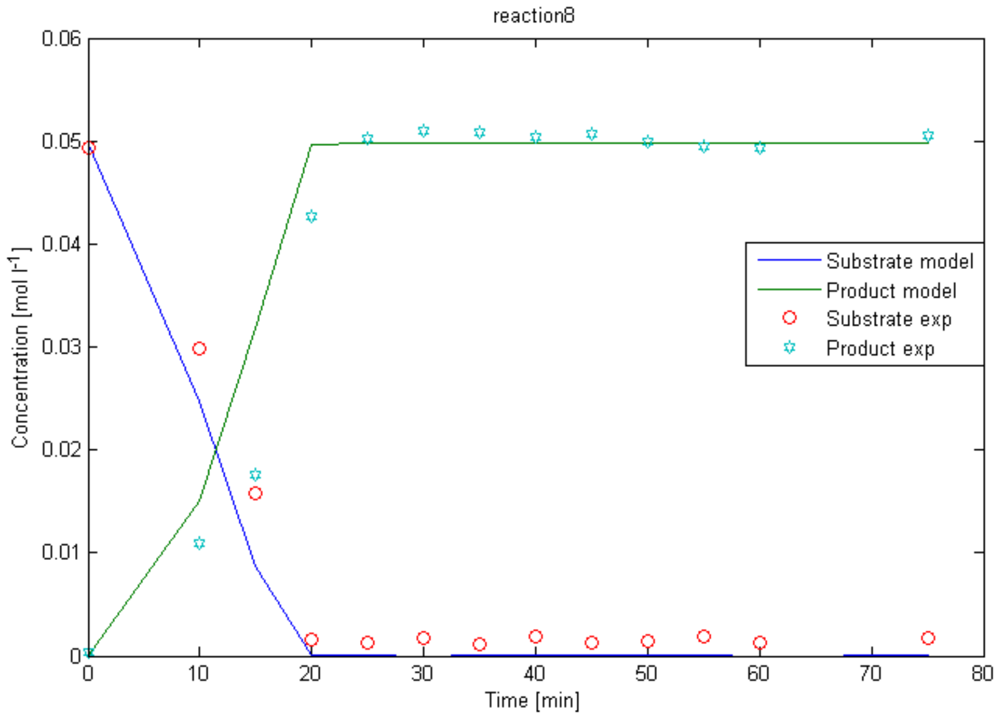
reaction8
Concentration [mol l-1]
Time [min]
Substrate model
Product model
Substrate exp
Product exp

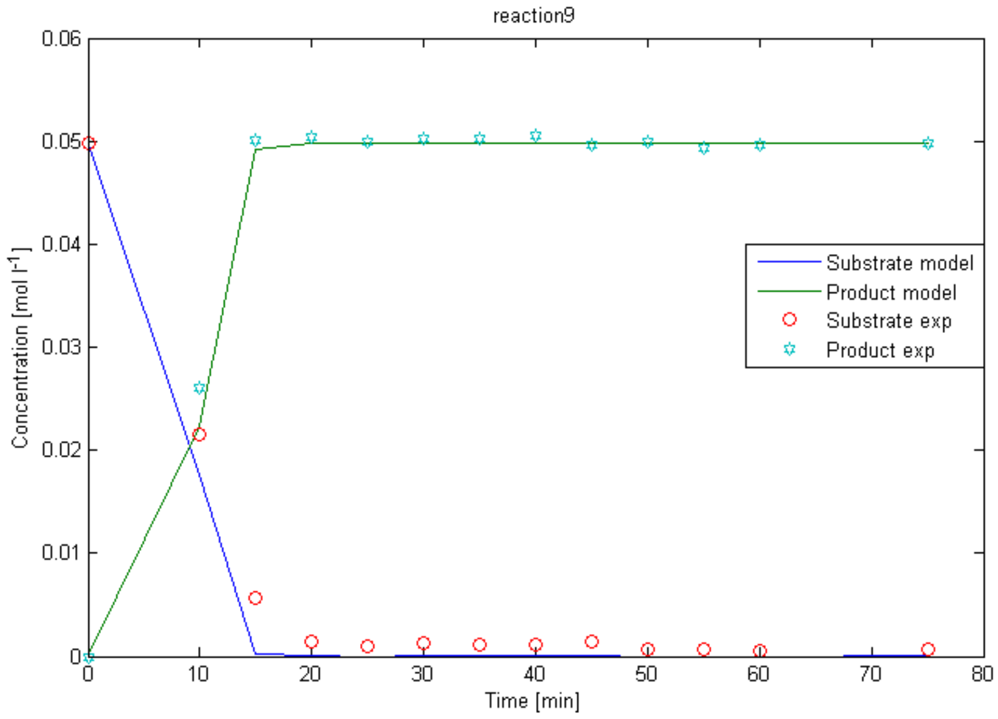
reaction9
Concentration [mol l-1]
Time [min]
Substrate model
Product model
Substrate exp
Product exp

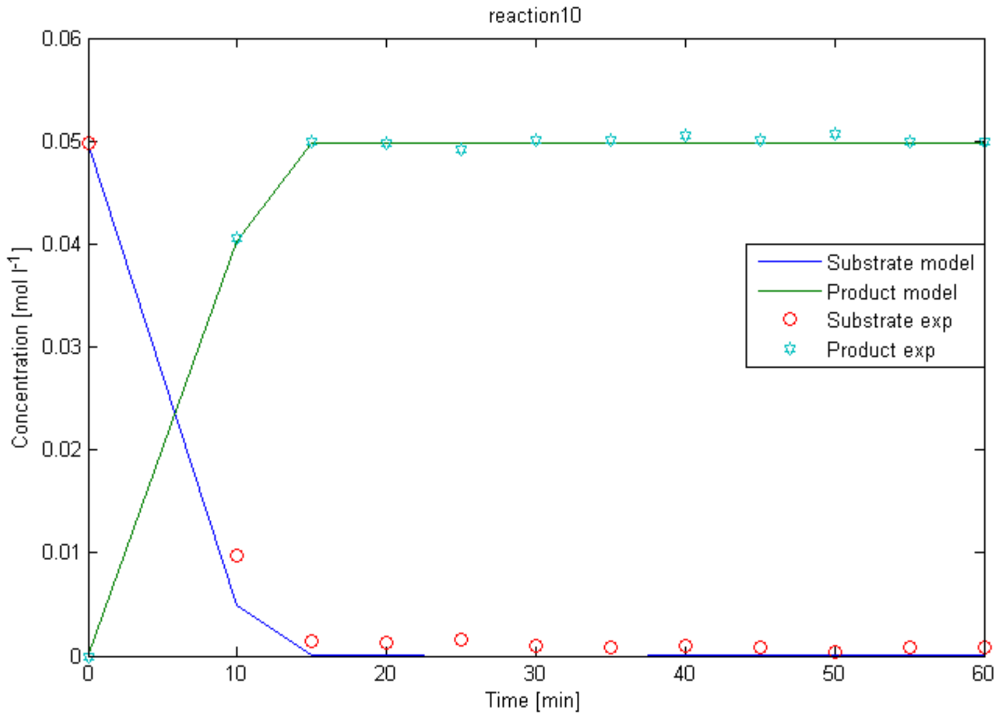
reaction10
0.06
0.05
0.04
0.03
0.02
0.01
0
Concentration [mol l-1]
0
10
20
30
40
50
60
Time [min]
Substrate model
Product model
Substrate exp
Product exp

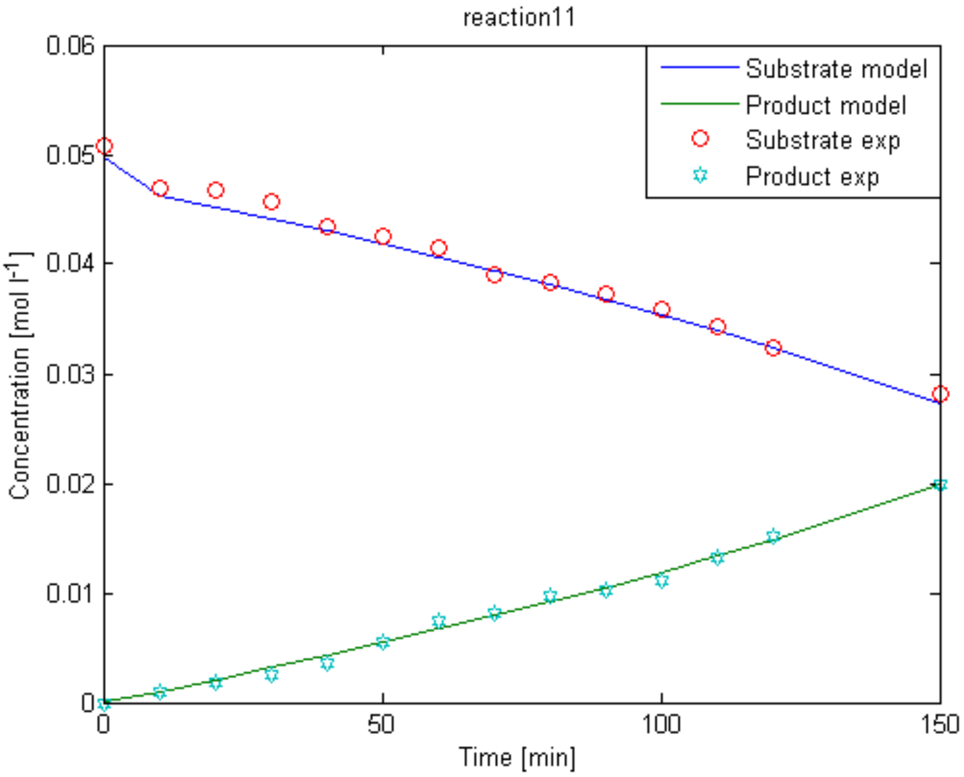
reaction11
0.06
0.05
0.04
0.03
0.02
0.01
0
Concentration [mol l-1]
0
50
100
150
Time [min]
Substrate model
Product model
Substrate exp
Product exp

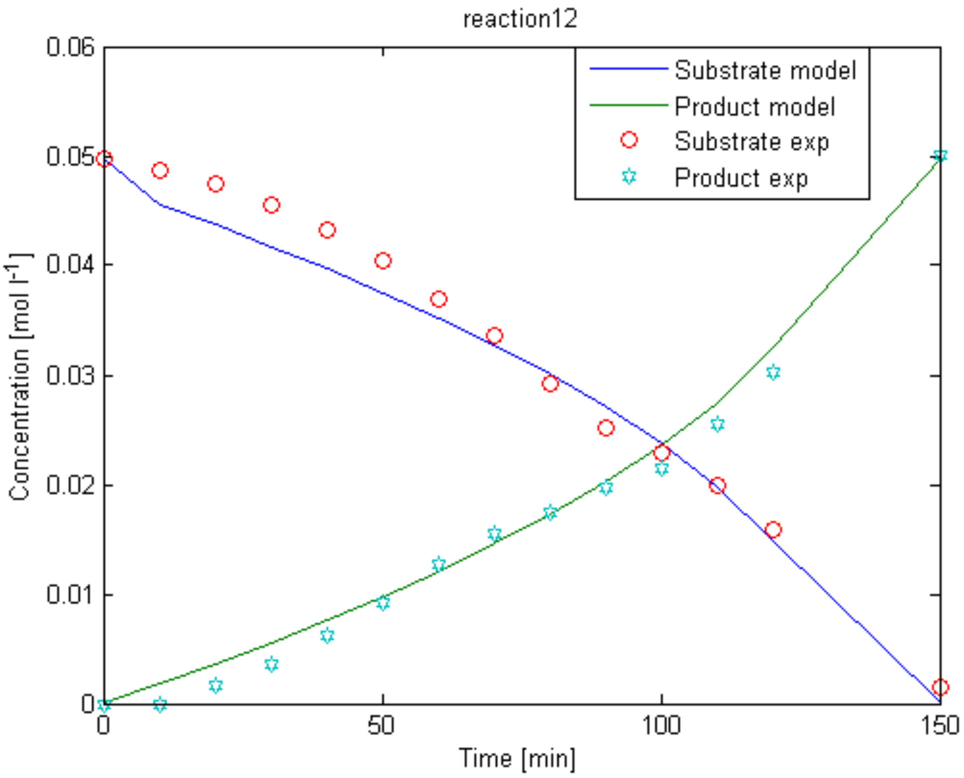
reaction12
Substrate model
Product model
Substrate exp
Product exp
Concentration [mol l-1]
Time [min]

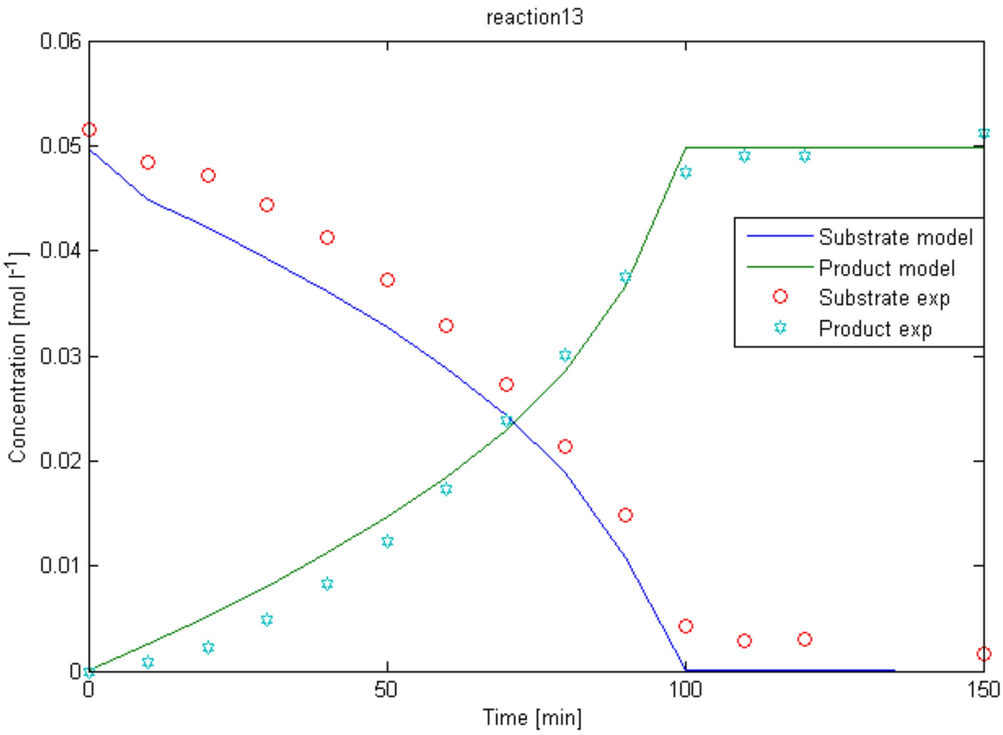
reaction13
Substrate model
Product model
Substrate exp
Product exp
Concentration [mol l-1]
Time [min]

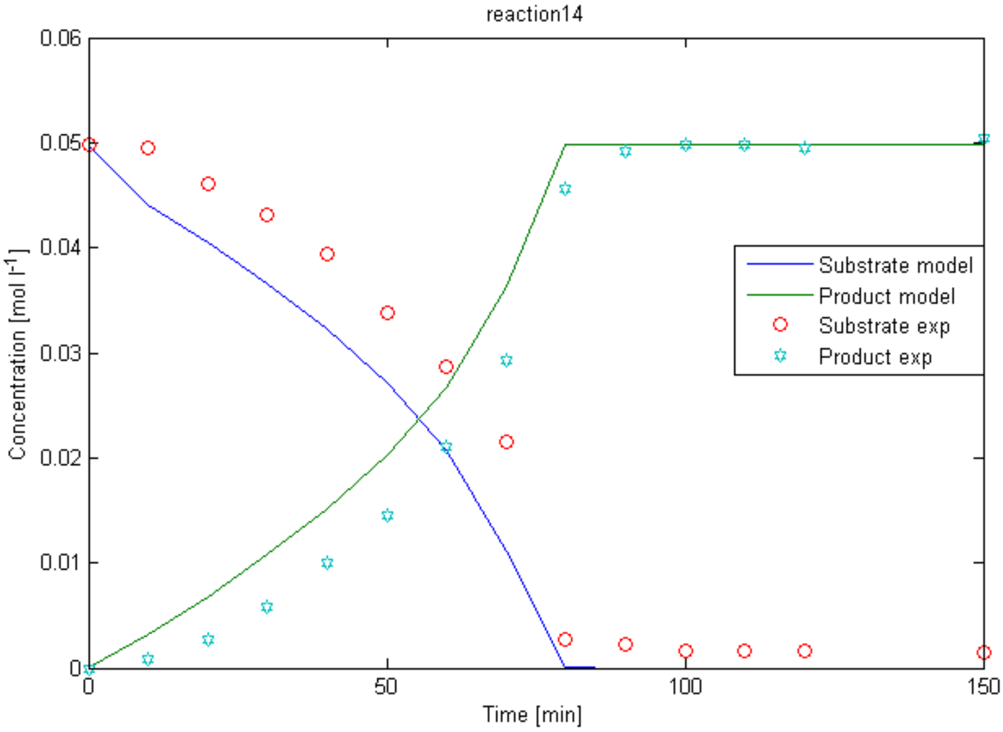
reaction14
Concentration [mol l-1]
Time [min]
Substrate model
Product model
Substrate exp
Product exp

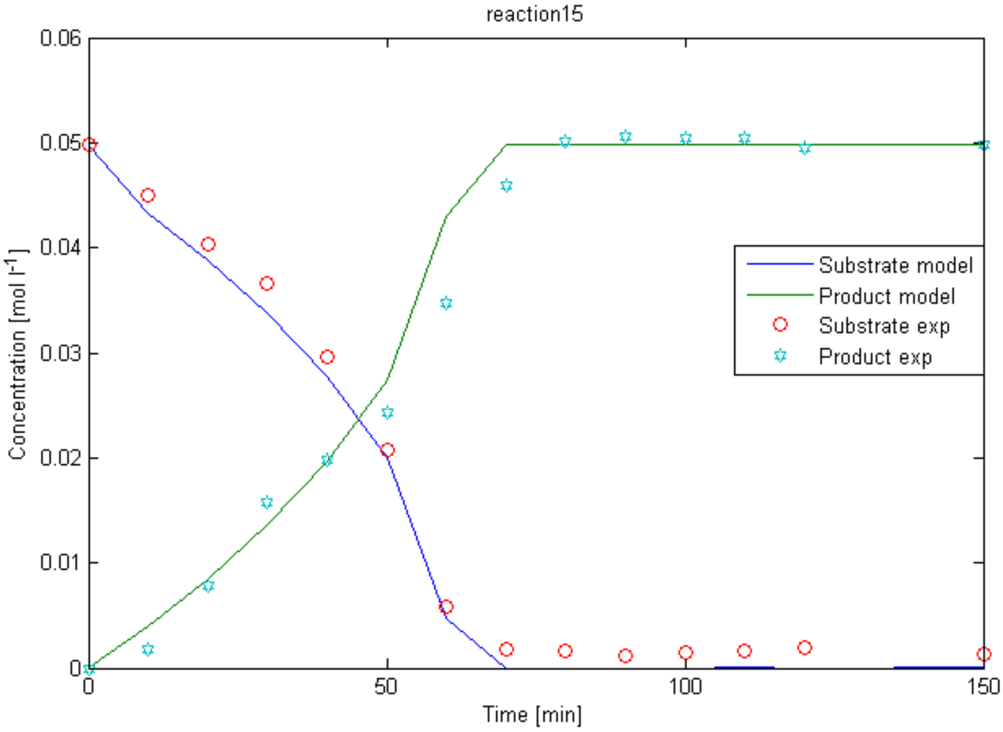
reaction15
Concentration [mol l-1]
Time [min]
Substrate model
Product model
Substrate exp
Product exp

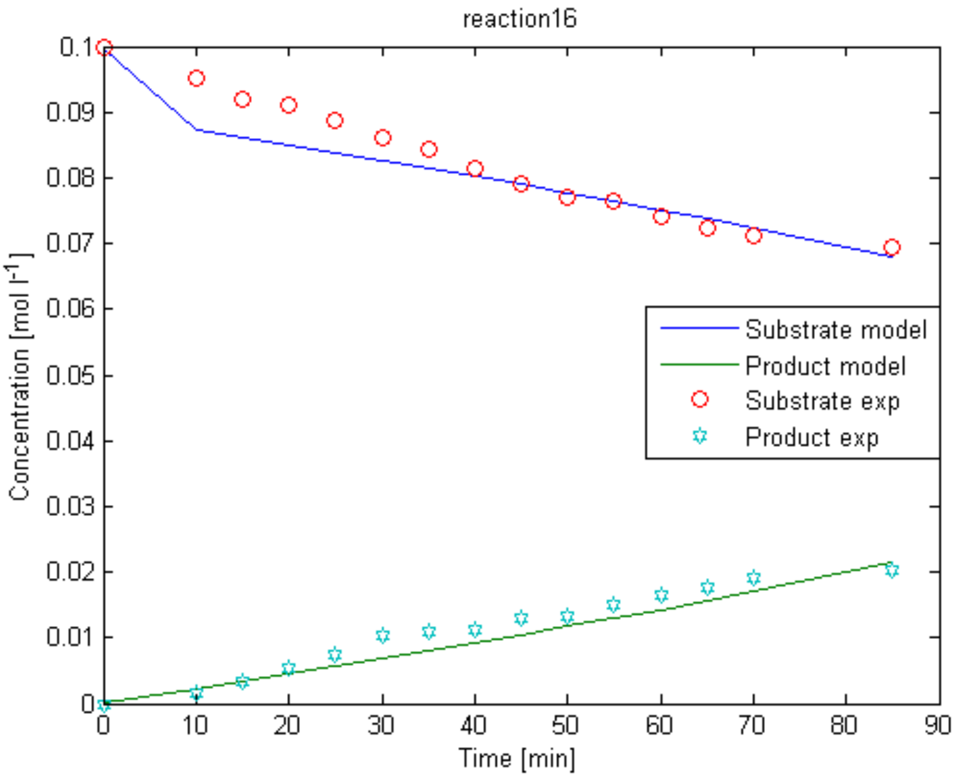

reaction16
0.1
0.09
0.08
0.07
0.06
0.05
0.04
0.03
0.02
0.01
0
Concentration [mol l-1]
0 10 20 30 40 50 60 70 80 90
Time [min]
Substrate model
Product model
Substrate exp
Product exp

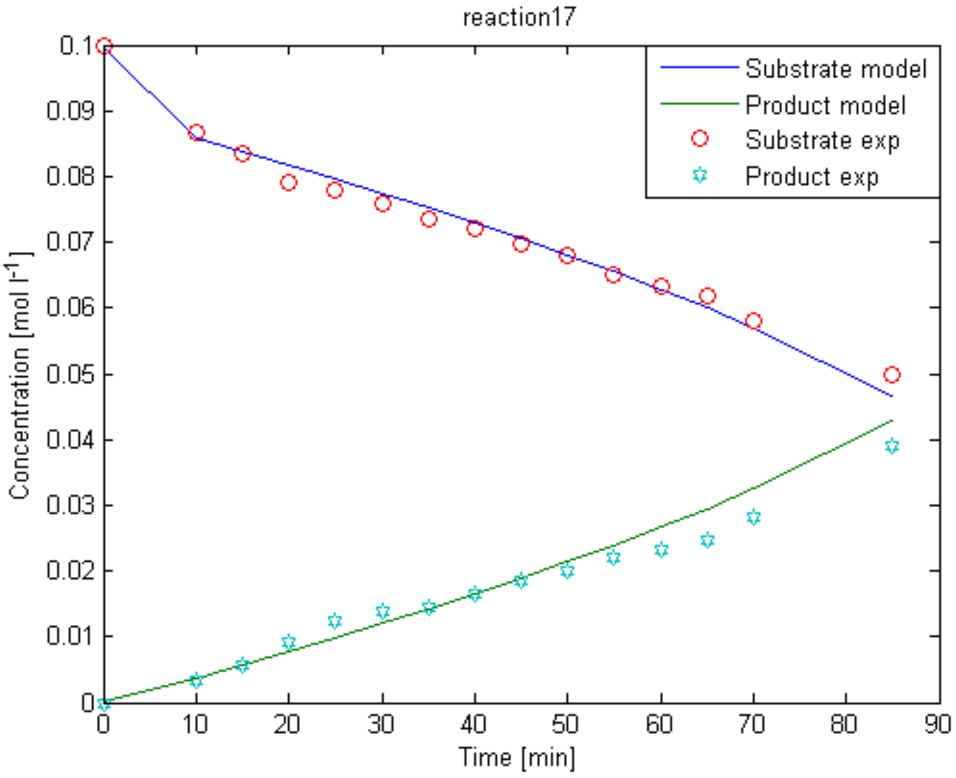

reaction17
0.1
0.09
0.08
0.07
0.06
0.05
0.04
0.03
0.02
0.01
0
Concentration [mol l-1]
0 10 20 30 40 50 60 70 80 90
Time [min]
Substrate model
Product model
Substrate exp
Product exp

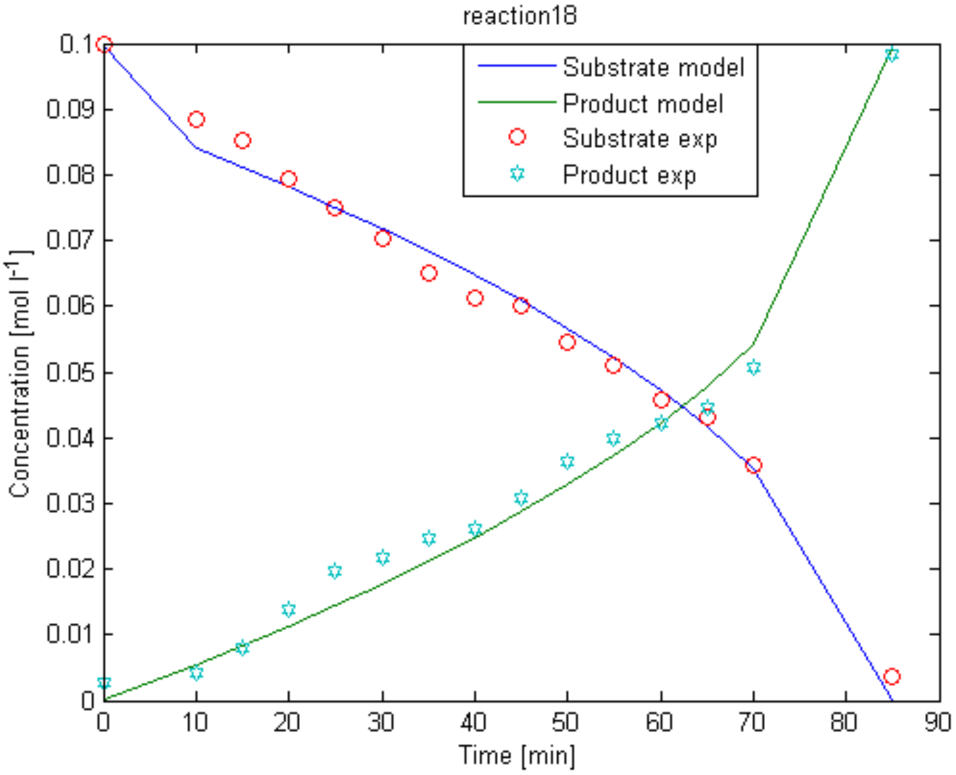
reaction18
Substrate model
Product model
Substrate exp
Product exp
Concentration [mol l^{-1}]
Time [min]
0.1
0.09
0.08
0.07
0.06
0.05
0.04
0.03
0.02
0.01
0
0
10
20
30
40
50
60
70
80
90

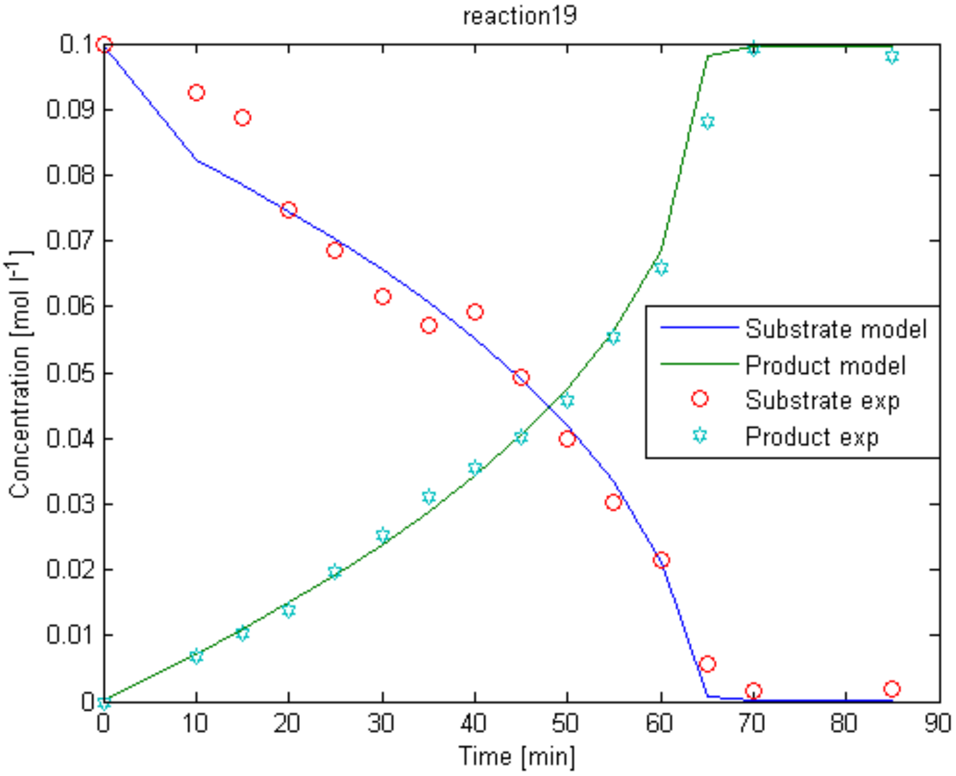
reaction19
Substrate model
Product model
Substrate exp
Product exp
Concentration [mol l^{-1}]
Time [min]
0.1
0.09
0.08
0.07
0.06
0.05
0.04
0.03
0.02
0.01
0
0
10
20
30
40
50
60
70
80
90

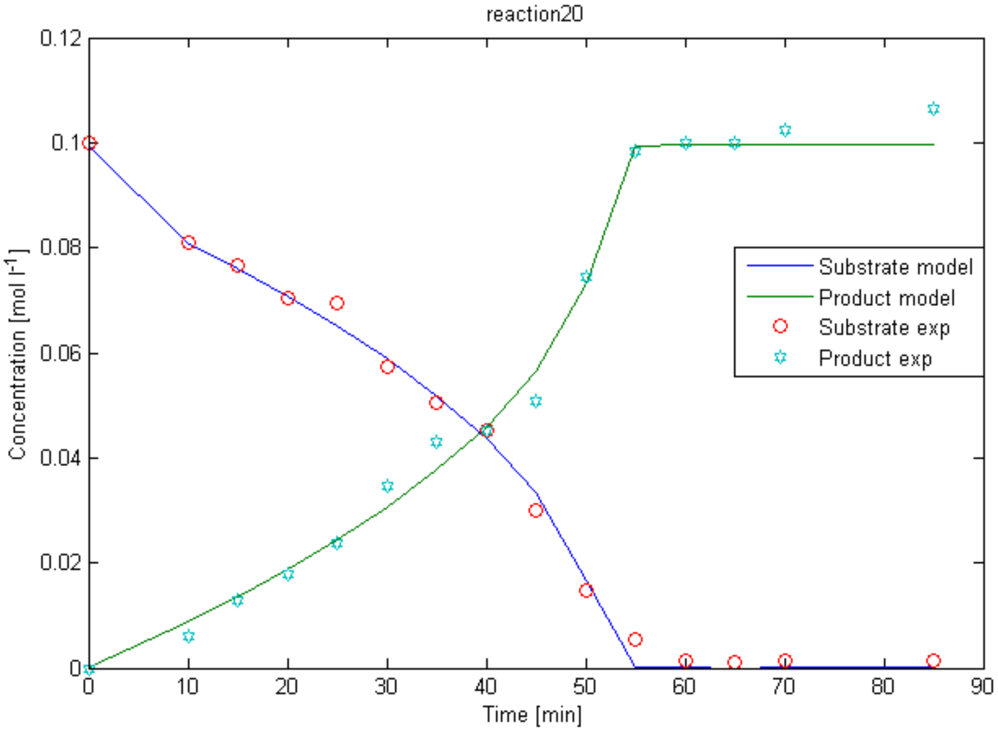
reaction20
0.12
0.1
0.08
0.06
0.04
0.02
0
Concentration [mol l-1]
0
10
20
30
40
50
60
70
80
90
Time [min]
Substrate model
Product model
Substrate exp
Product exp

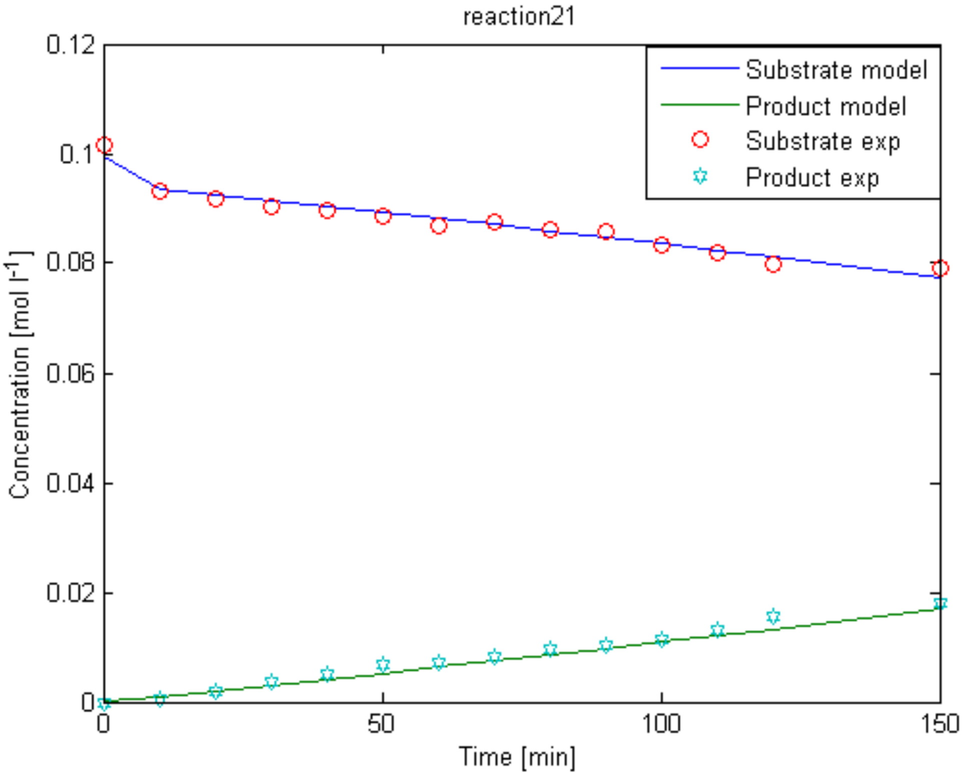
reaction21
0.12
0.1
0.08
0.06
0.04
0.02
0
Concentration [mol l-1]
0
50
100
150
Time [min]
Substrate model
Product model
Substrate exp
Product exp

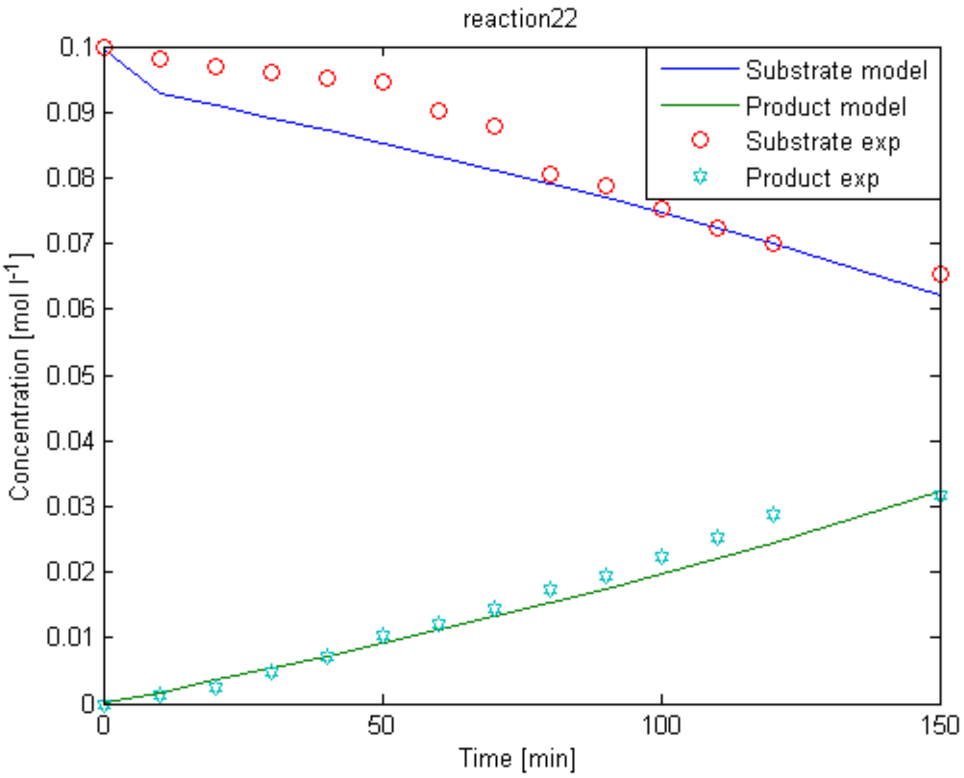
reaction22
Substrate model
Product model
Substrate exp
Product exp
Concentration [mol l-1]
Time [min]
0.1
0.09
0.08
0.07
0.06
0.05
0.04
0.03
0.02
0.01
0
50
100
150

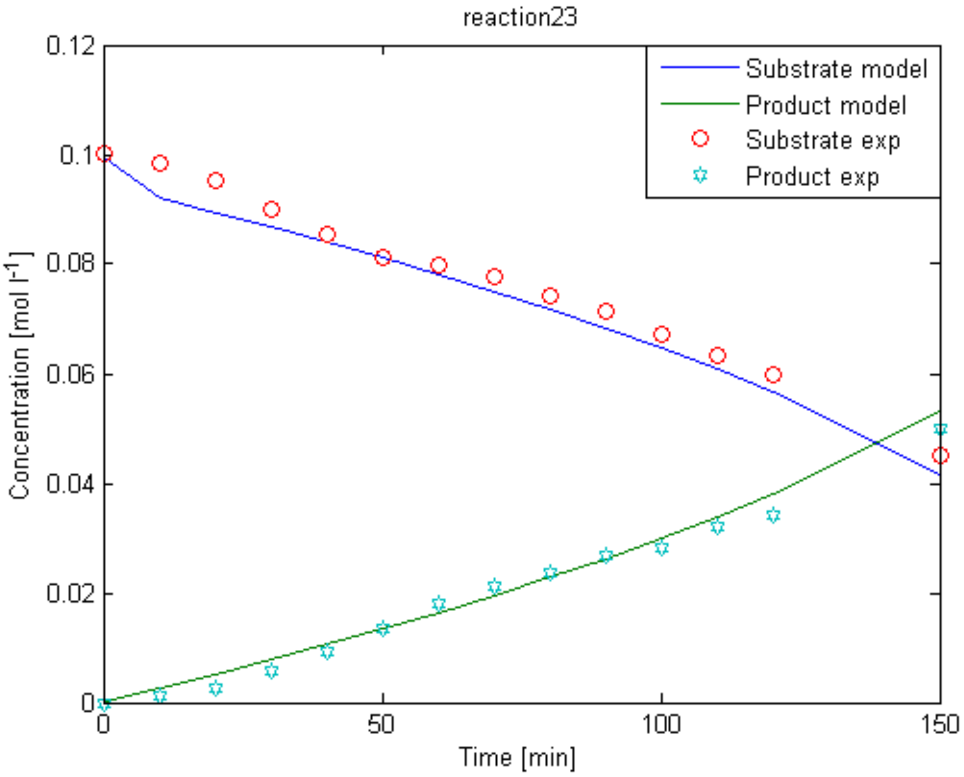
reaction23
Substrate model
Product model
Substrate exp
Product exp
Concentration [mol l-1]
Time [min]
0.12
0.1
0.08
0.06
0.04
0.02
0
50
100
150

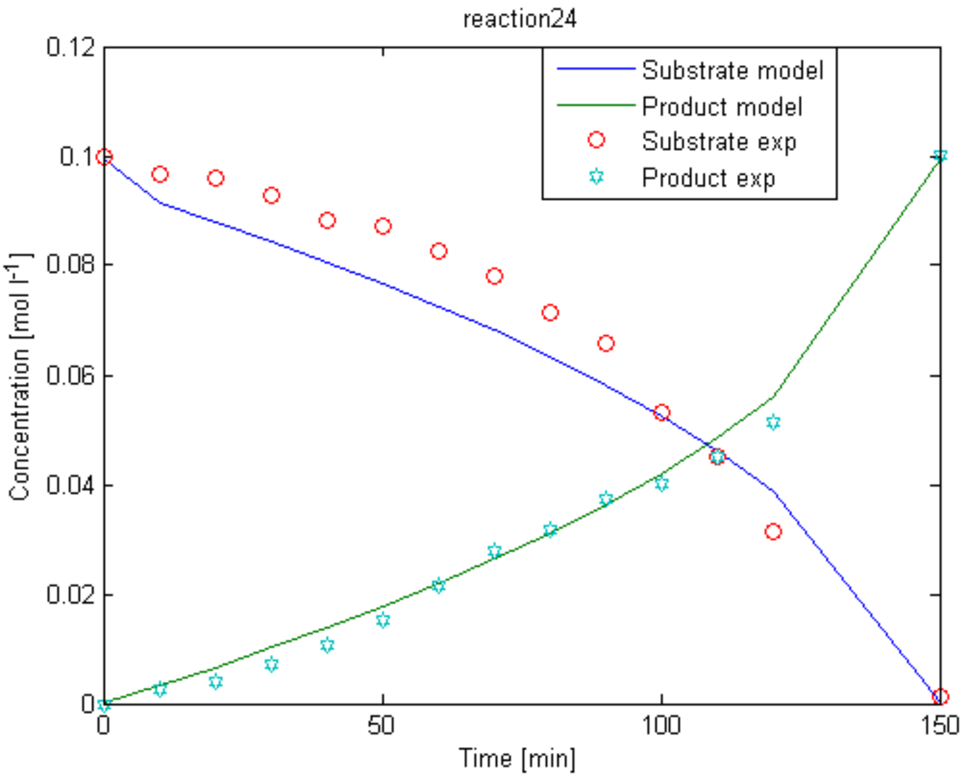
reaction24
Substrate model
Product model
Substrate exp
Product exp
Concentration [mol l-1]
Time [min]
0
0.02
0.04
0.06
0.08
0.1
0.12
0
50
100
150

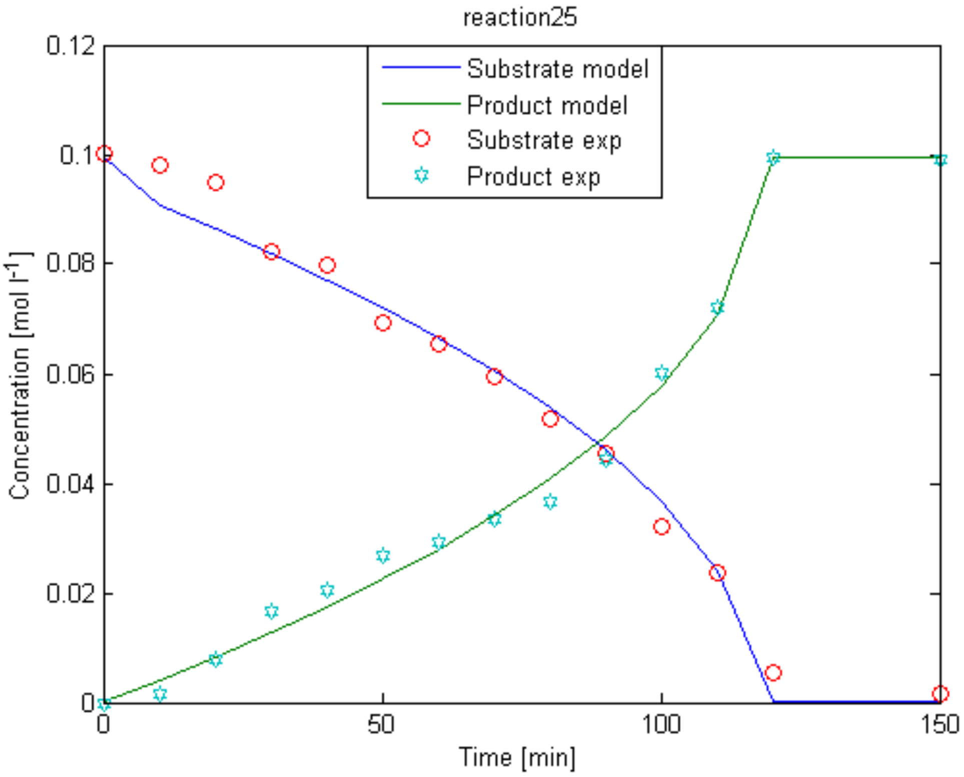
reaction25
Substrate model
Product model
Substrate exp
Product exp
Concentration [mol l-1]
Time [min]
0
0.02
0.04
0.06
0.08
0.1
0.12
0
50
100
150

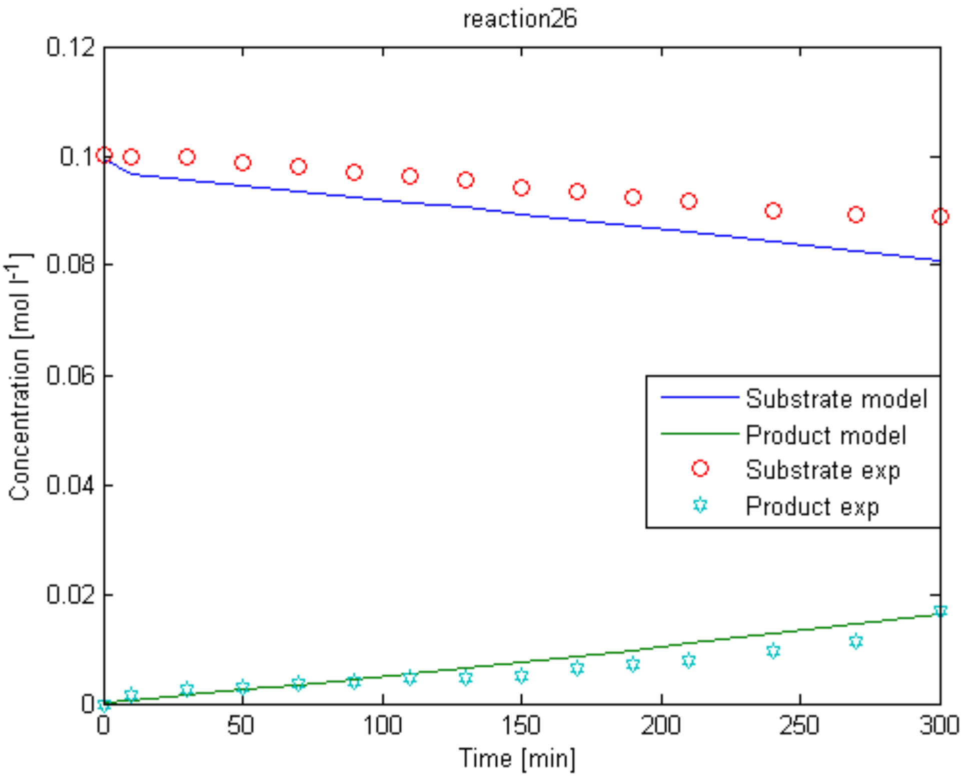
reaction26
Concentration [mol l-1]
Time [min]
Substrate model
Product model
Substrate exp
Product exp
0.12
0.1
0.08
0.06
0.04
0.02
0
0
50
100
150
200
250
300

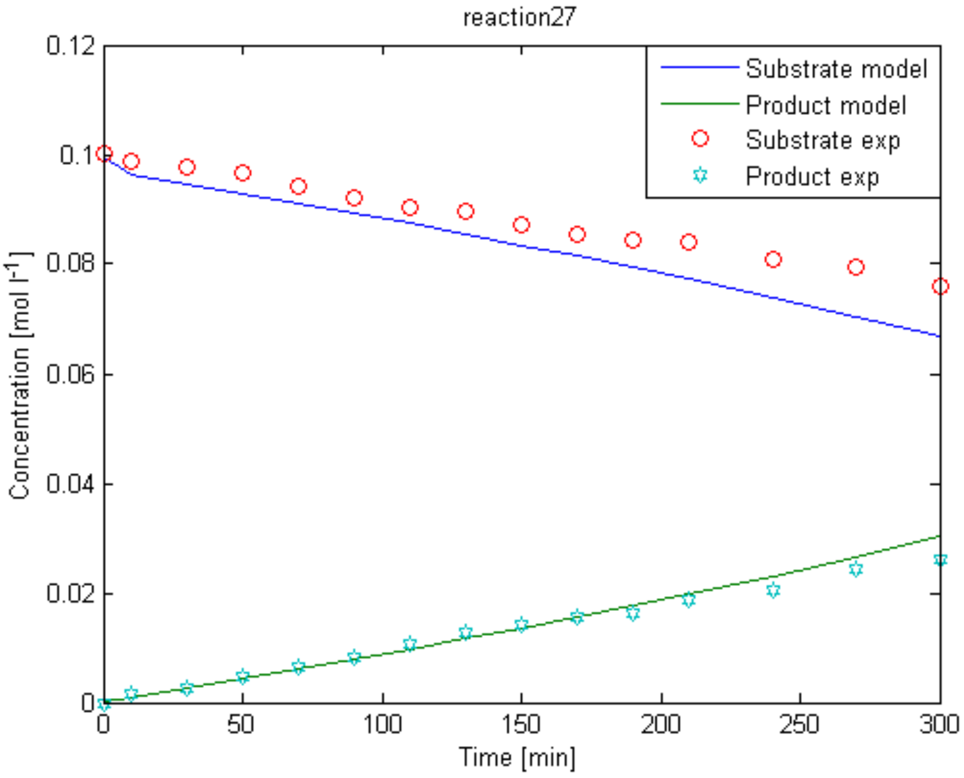
reaction27
Concentration [mol l-1]
Time [min]
Substrate model
Product model
Substrate exp
Product exp
0.12
0.1
0.08
0.06
0.04
0.02
0
0
50
100
150
200
250
300

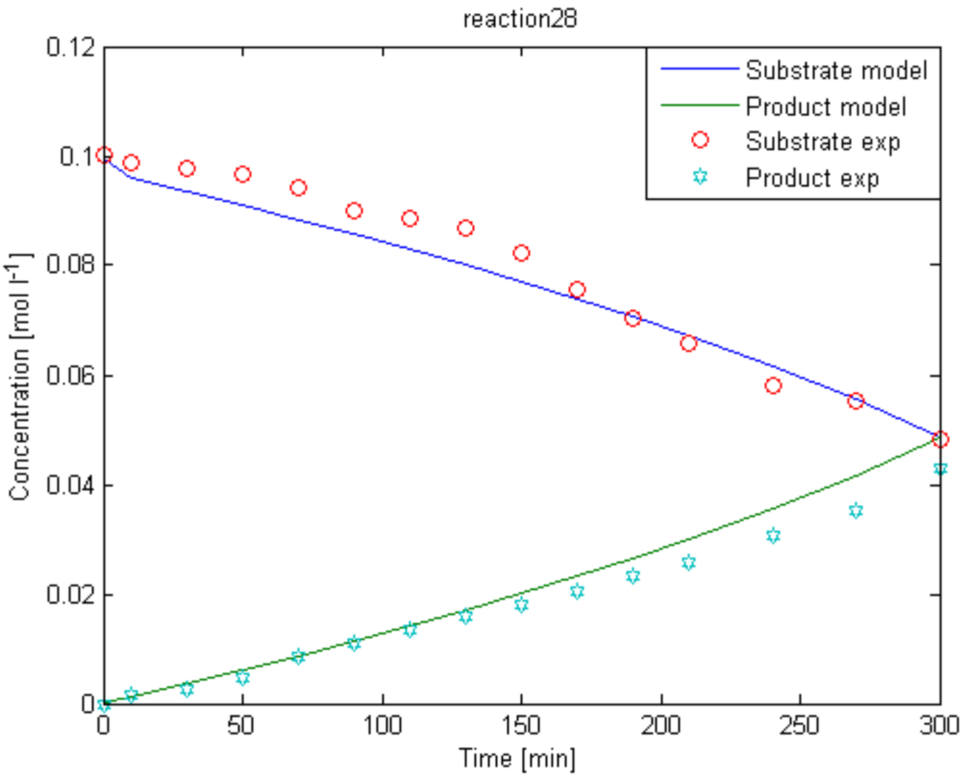
reaction28
Substrate model
Product model
Substrate exp
Product exp
Concentration [mol l-1]
Time [min]

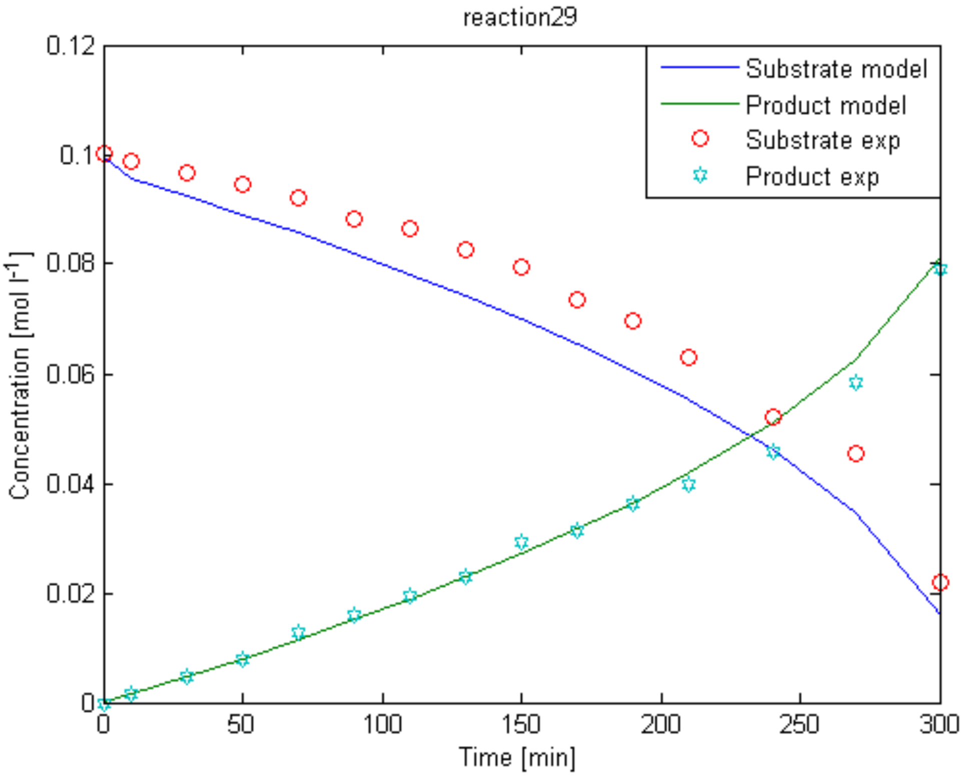
reaction29
Substrate model
Product model
Substrate exp
Product exp
Concentration [mol l-1]
Time [min]

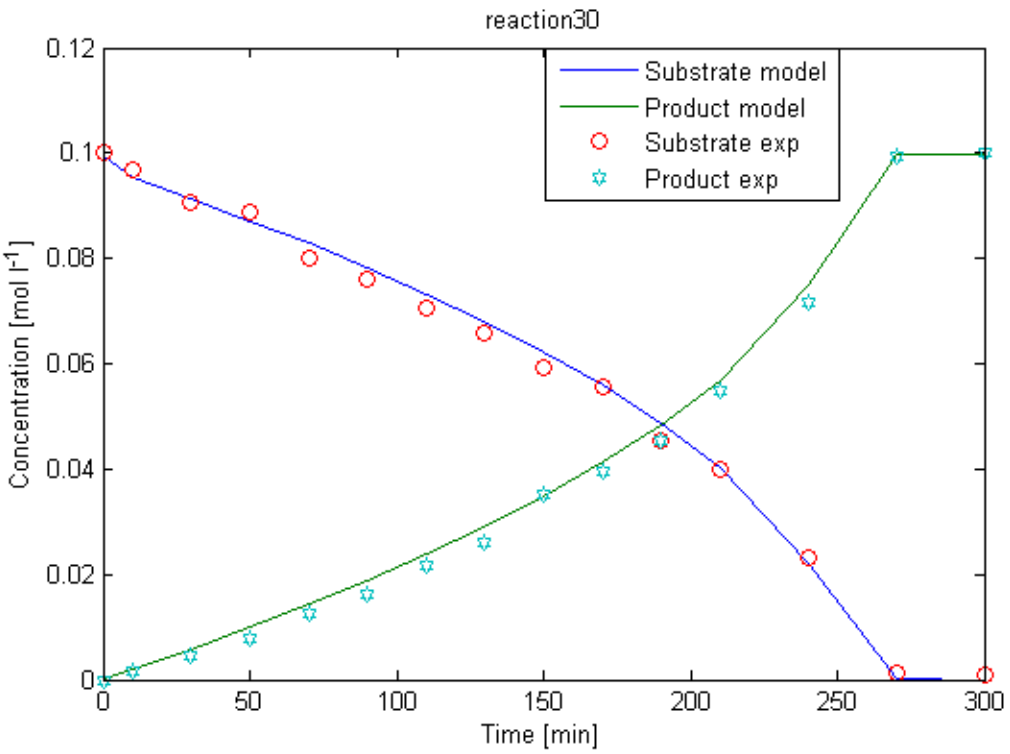
reaction30
Substrate model
Product model
Substrate exp
Product exp
Concentration [mol l-1]
Time [min]

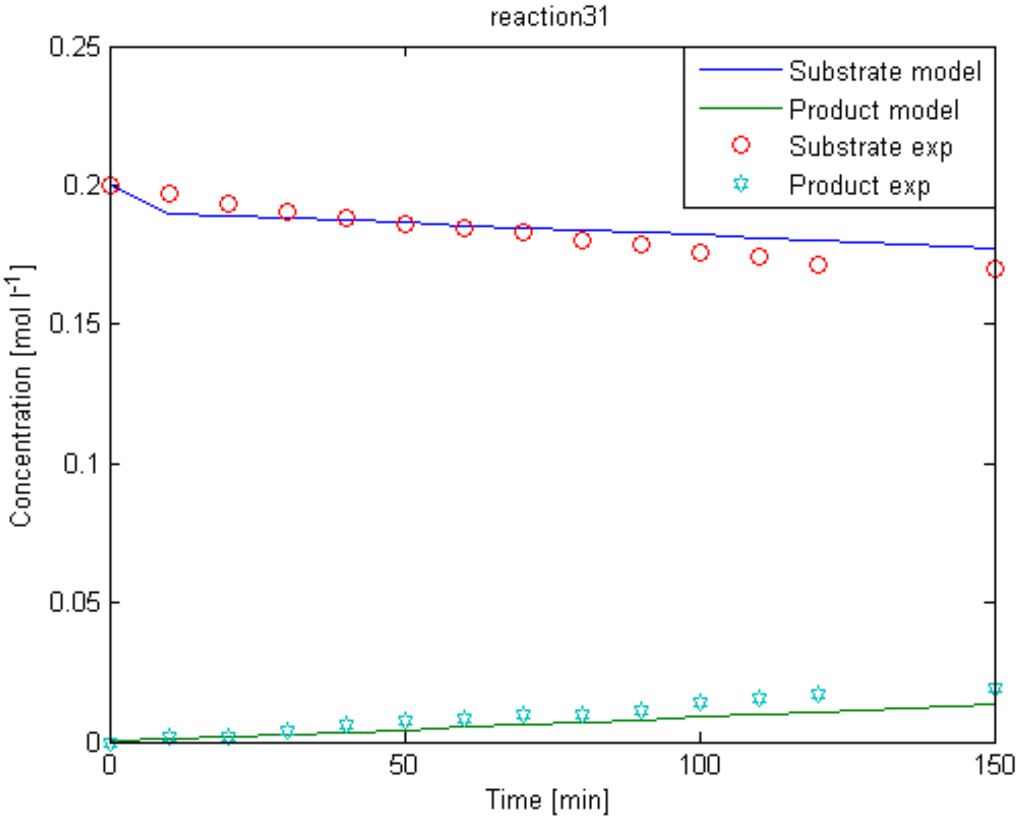
reaction31
Substrate model
Product model
Substrate exp
Product exp
Concentration [mol l-1]
Time [min]

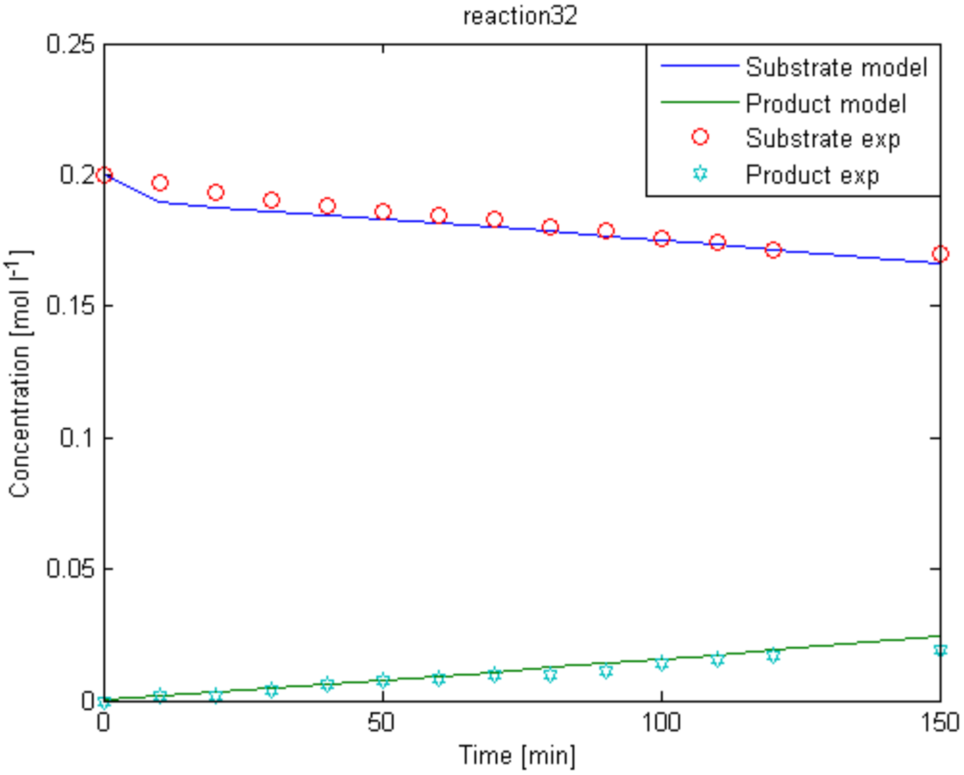
reaction32
Substrate model
Product model
Substrate exp
Product exp
Concentration [mol l-1]
Time [min]
0.25
0.2
0.15
0.1
0.05
0
50
100
150

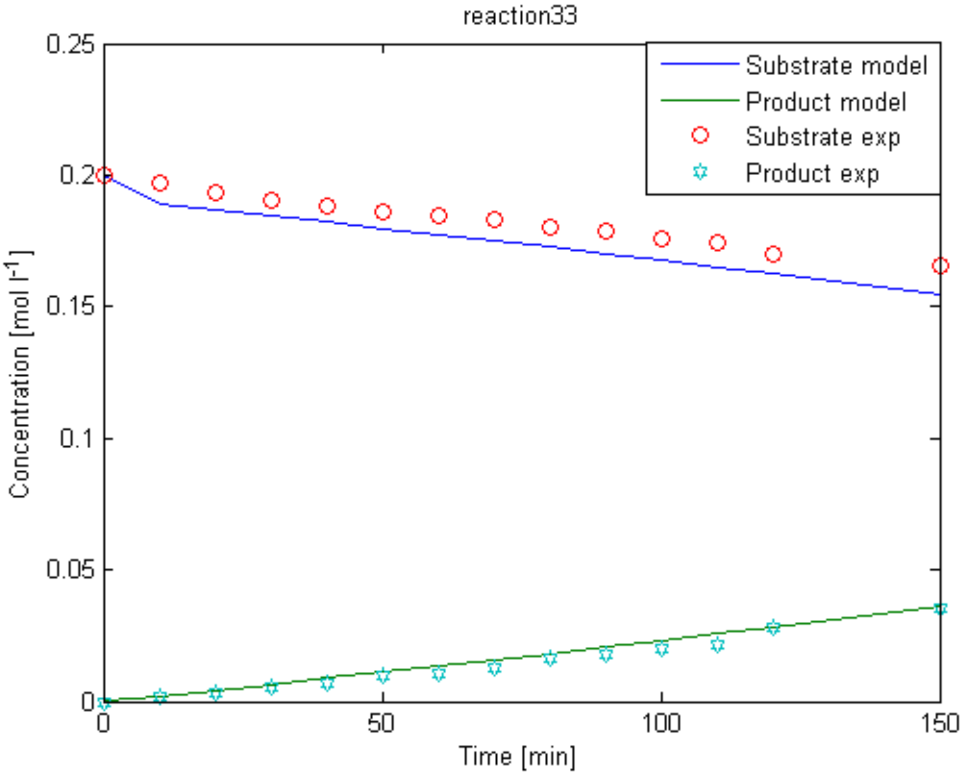
reaction33
Substrate model
Product model
Substrate exp
Product exp
Concentration [mol l-1]
Time [min]
0.25
0.2
0.15
0.1
0.05
0
50
100
150

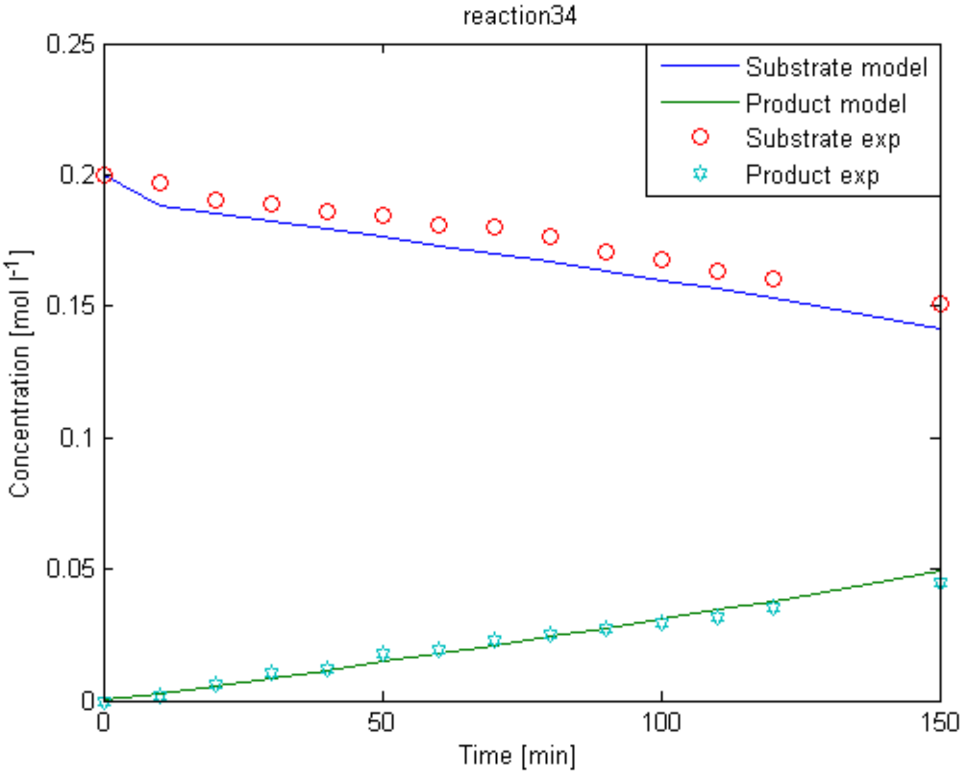
reaction34
Substrate model
Product model
Substrate exp
Product exp
Concentration [mol l-1]
Time [min]
0.25
0.2
0.15
0.1
0.05
0
50
100
150

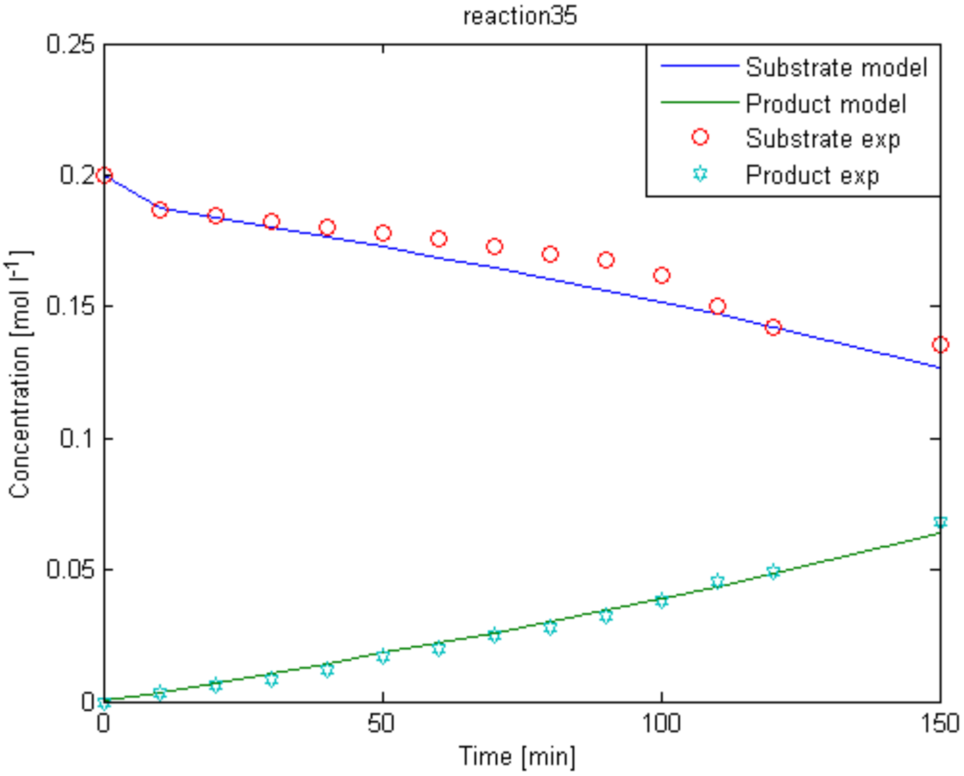
reaction35
Substrate model
Product model
Substrate exp
Product exp
Concentration [mol l-1]
Time [min]
0.25
0.2
0.15
0.1
0.05
0
50
100
150

7. NMR Spectra

1. 3,3,5,5-Tetramethylmorpholin-2-one; $CDCl_3$, 400MHz (^1H) 100MHz (^{13}C)

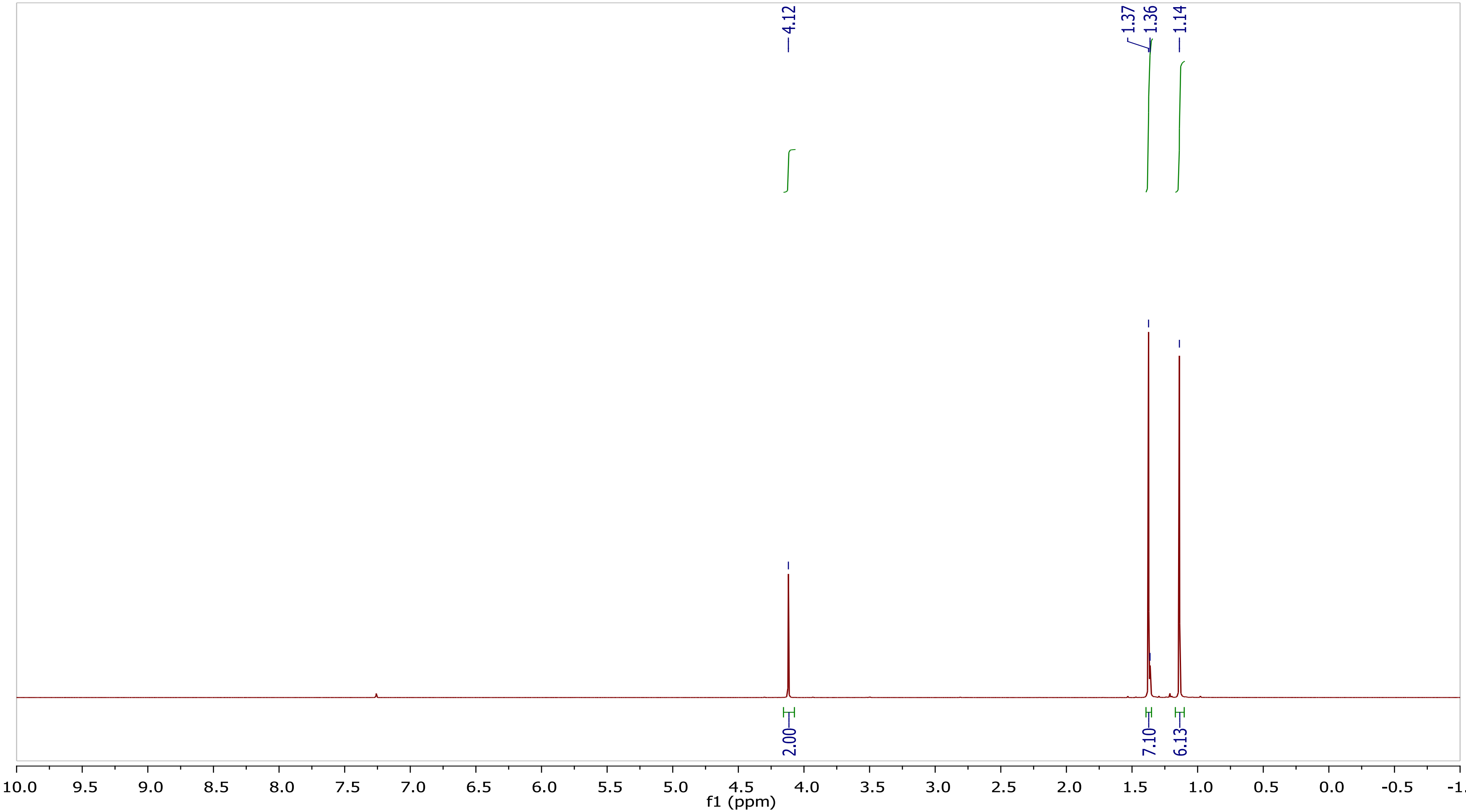

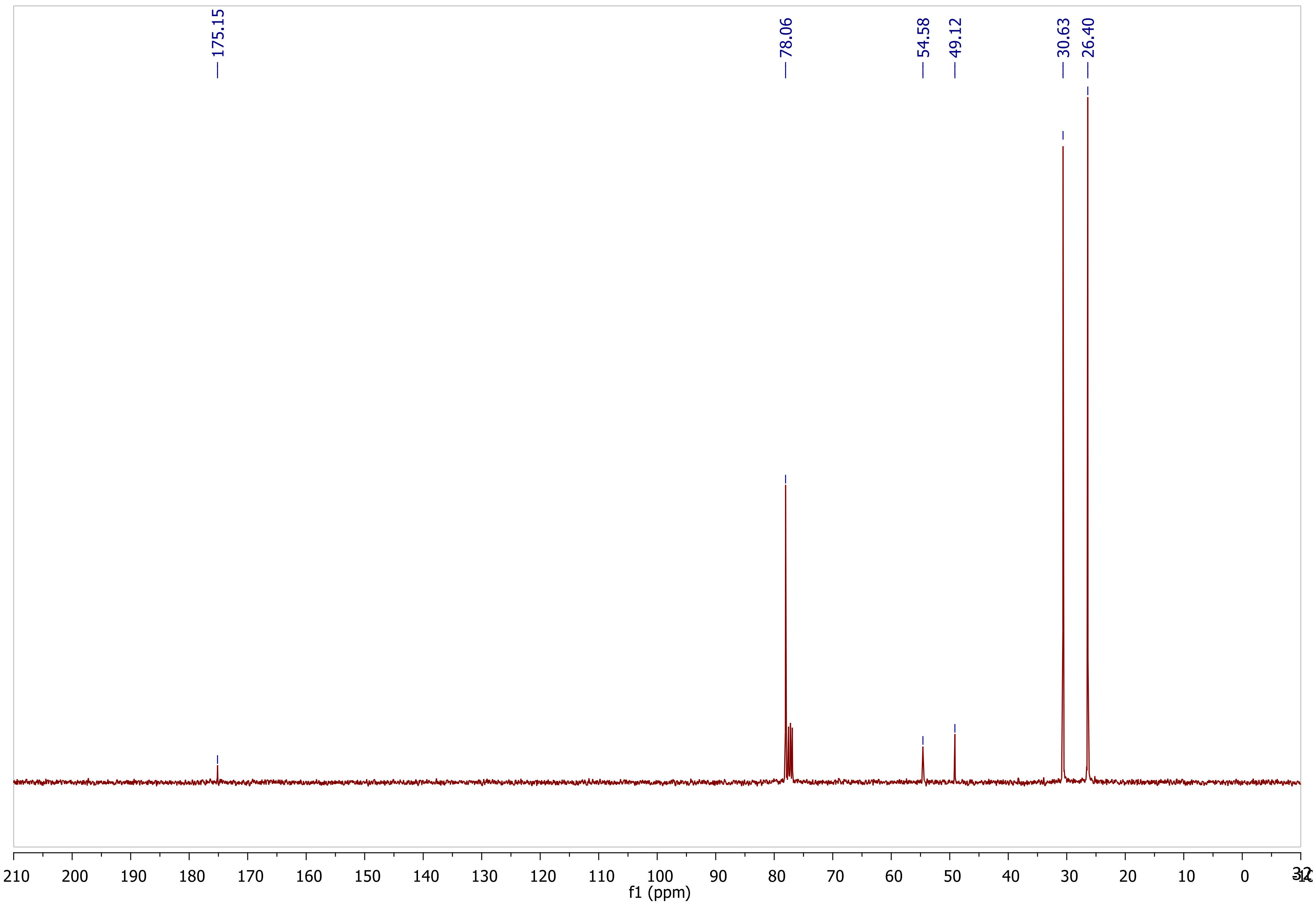

175.15
78.06
54.58
49.12
30.63
26.40
210 200 190 180 170 160 150 140 130 120 110 100 90 80 70 60 50 40 30 20 10 0
f1 (ppm)

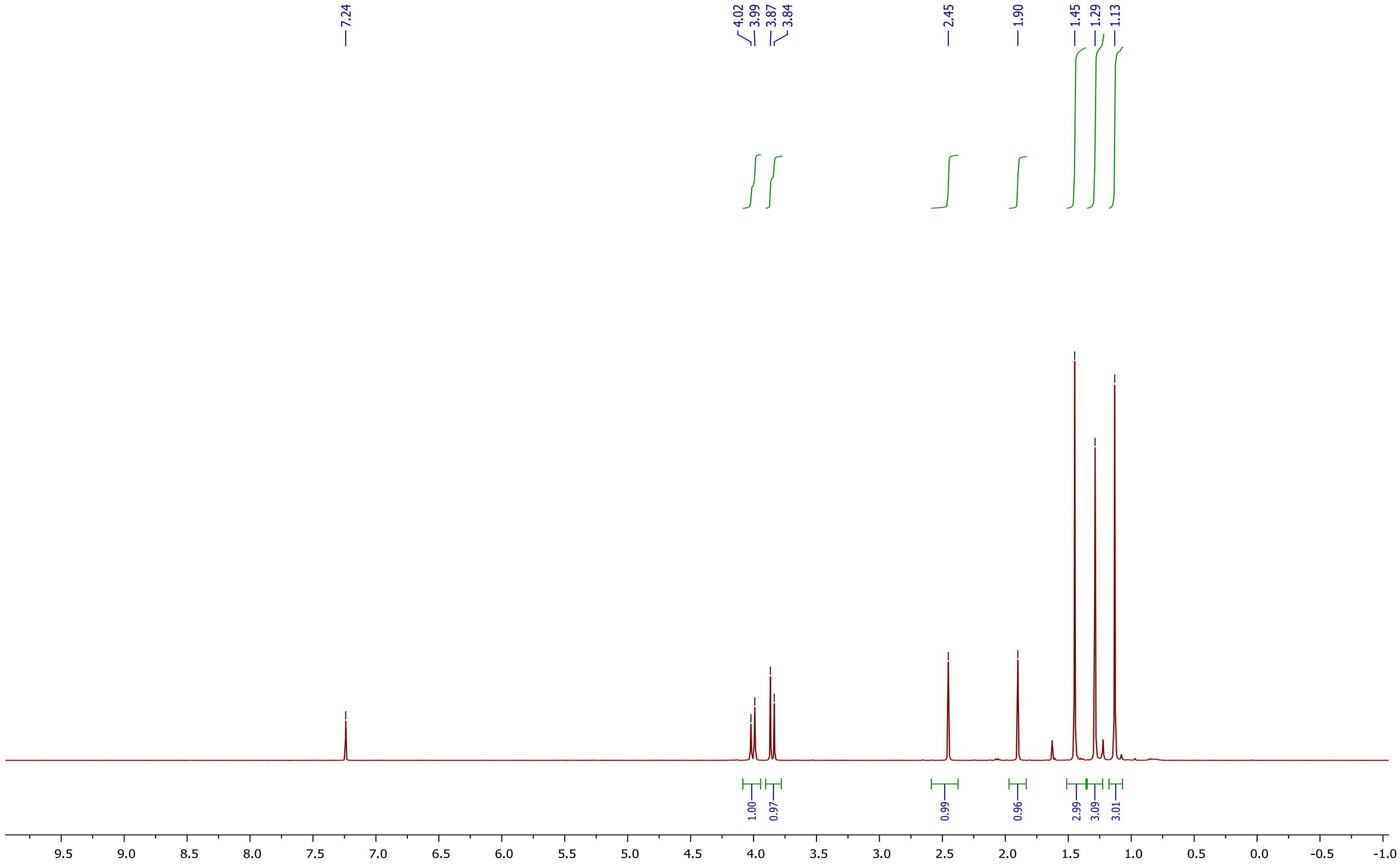

2 2,2,6-Trimethyl-4-oxa-1-azabicyclo[4.1.0]heptan-5-one $CDCl_3$, 400MHz (^{1}H) 100MHz (^{13}C)

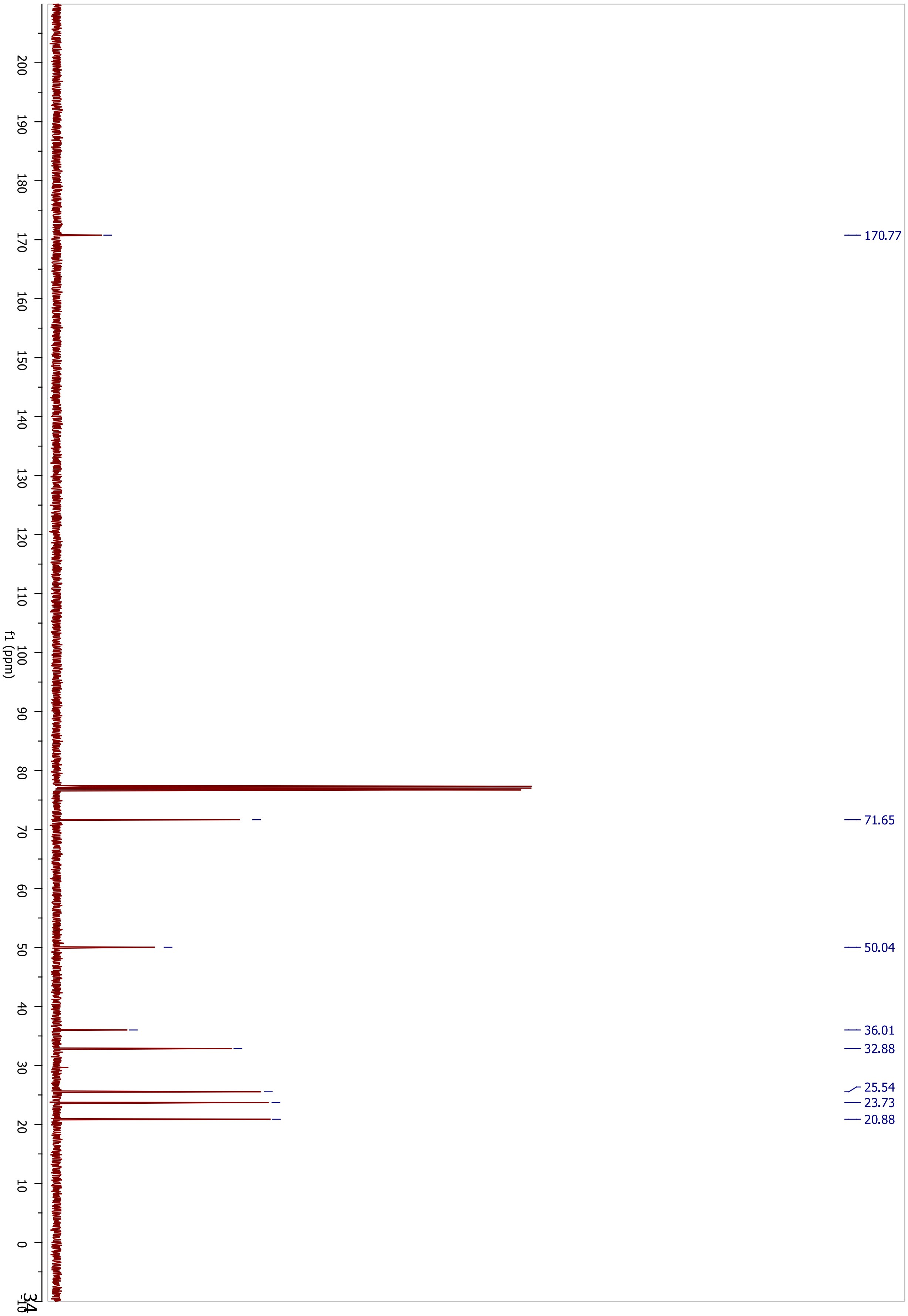
170.77
71.65
50.04
36.01
32.88
25.54
23.73
20.88
f1 (ppm)

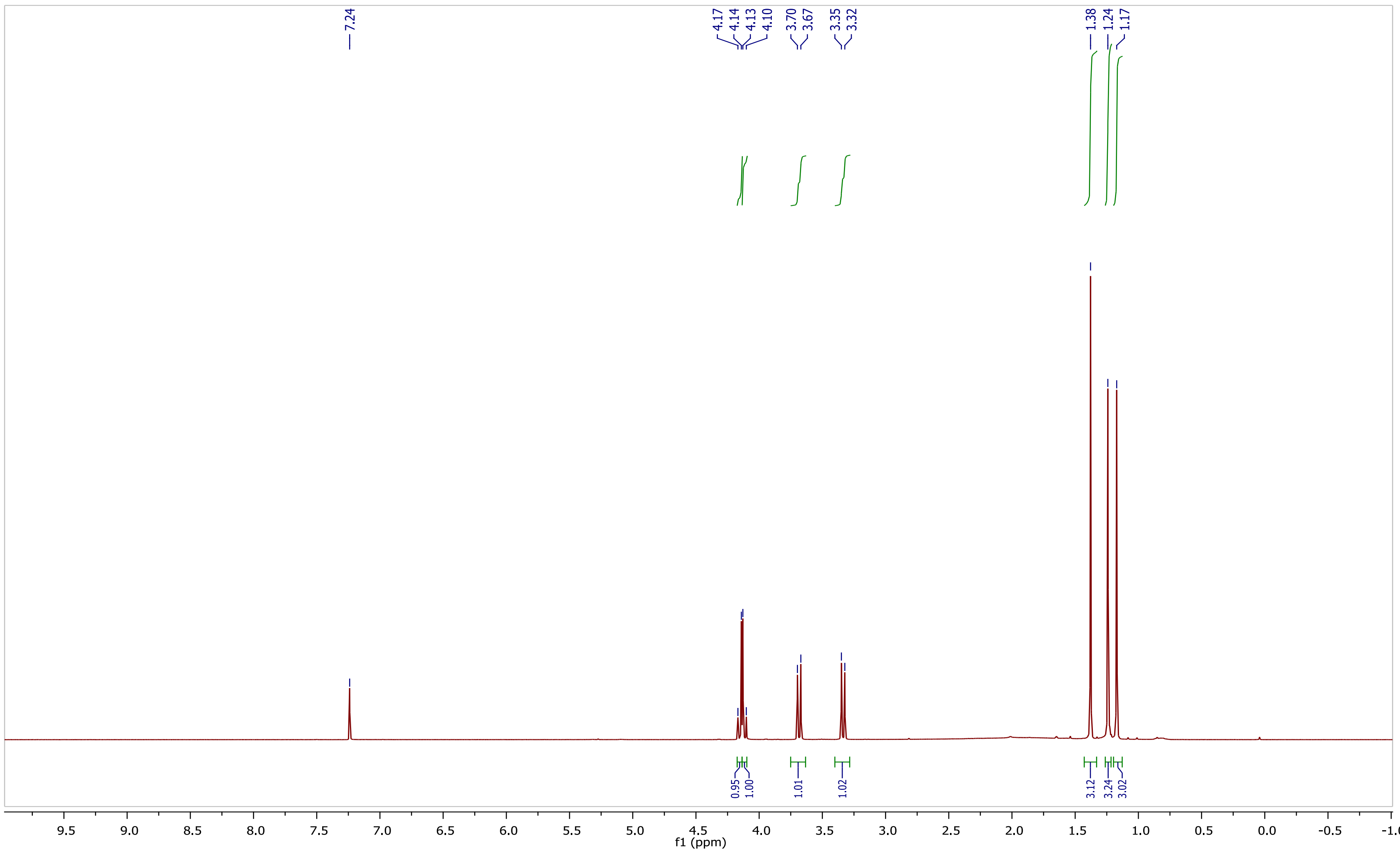

3a 3-(Hydroxymethyl)-3,5,5-trimethylmorpholin-2-one; $CDCl_3$, 400MHz (^{1}H) 100MHz (^{13}C)

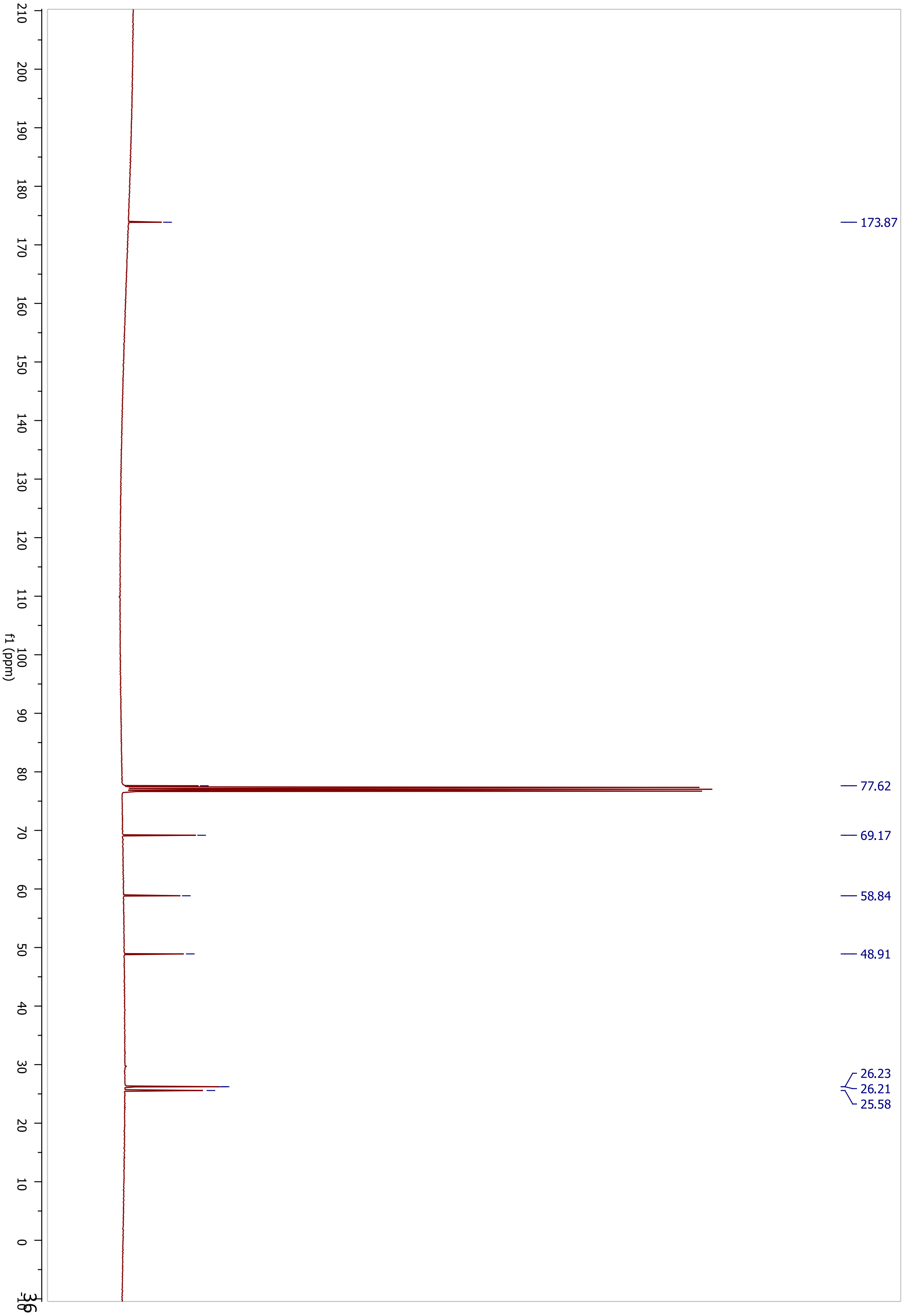
173.87
77.62
69.17
58.84
48.91
26.23
26.21
25.58
f1 (ppm)

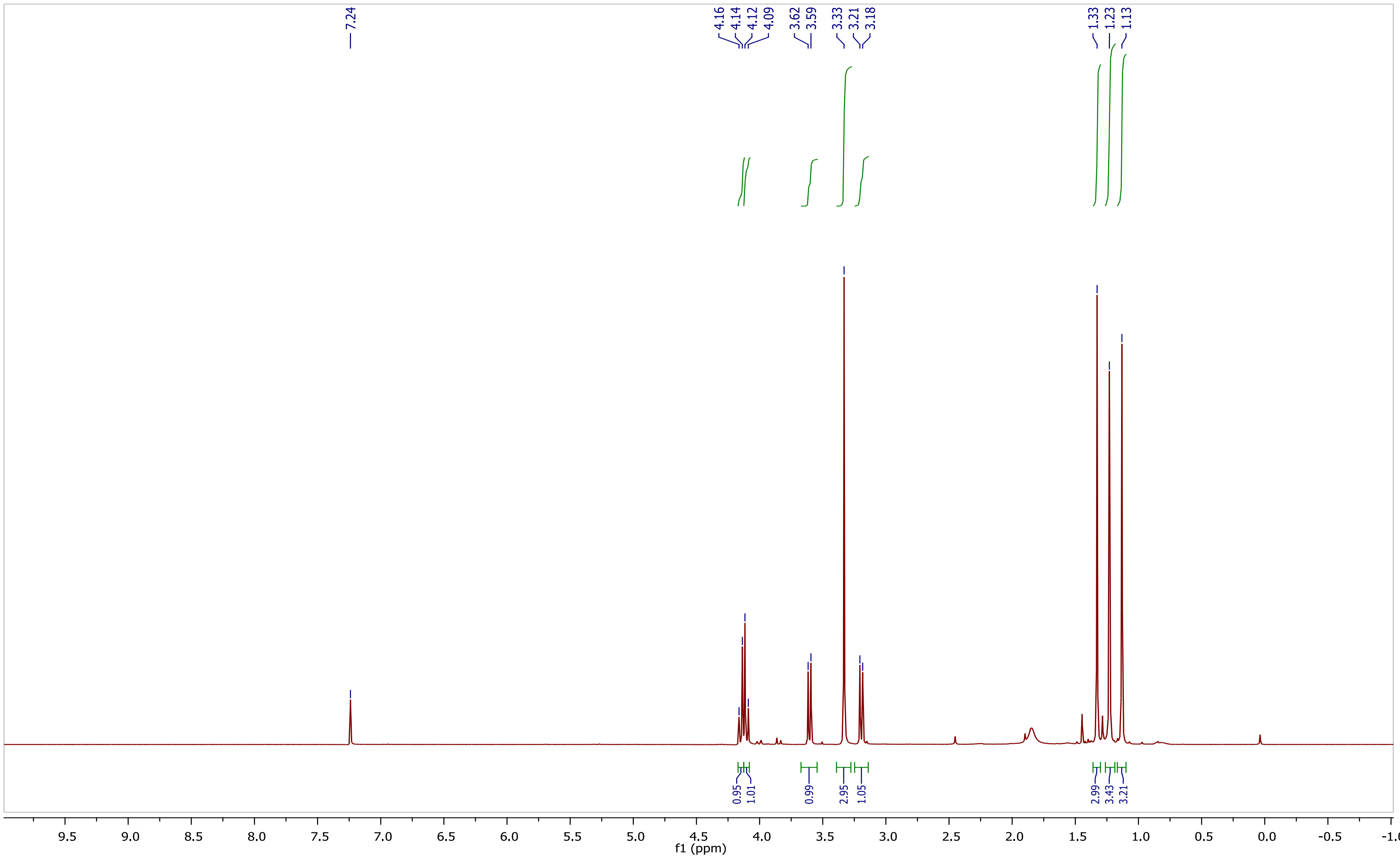

3b 3-(Methoxymethyl)-3,5,5-trimethylmorpholin-2-one; $CDCl_3$, 400MHz (^{1}H) 100MHz (^{13}C)

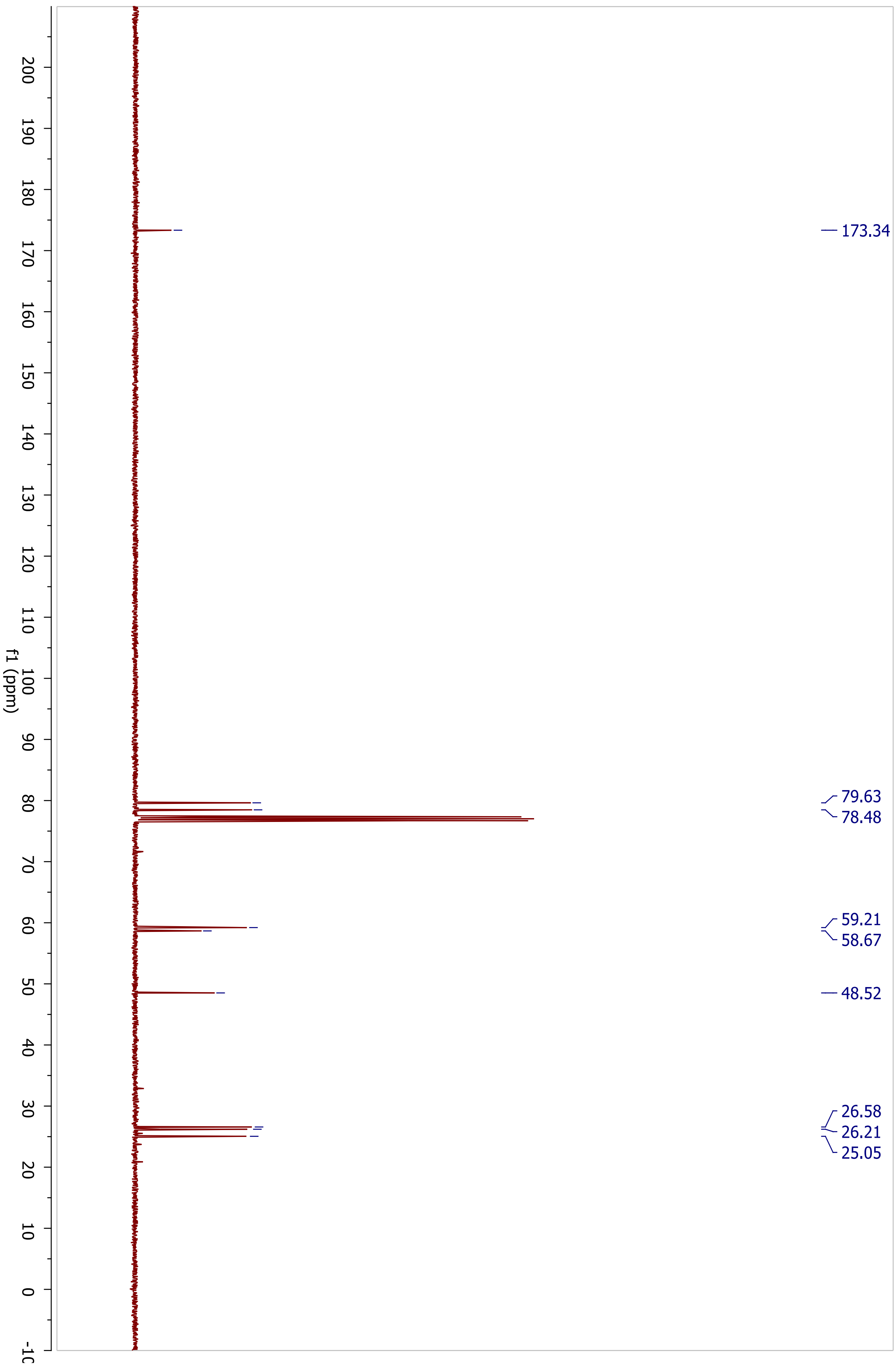
173.34
79.63
78.48
59.21
58.67
48.52
26.58
26.21
25.05
f1 (ppm)

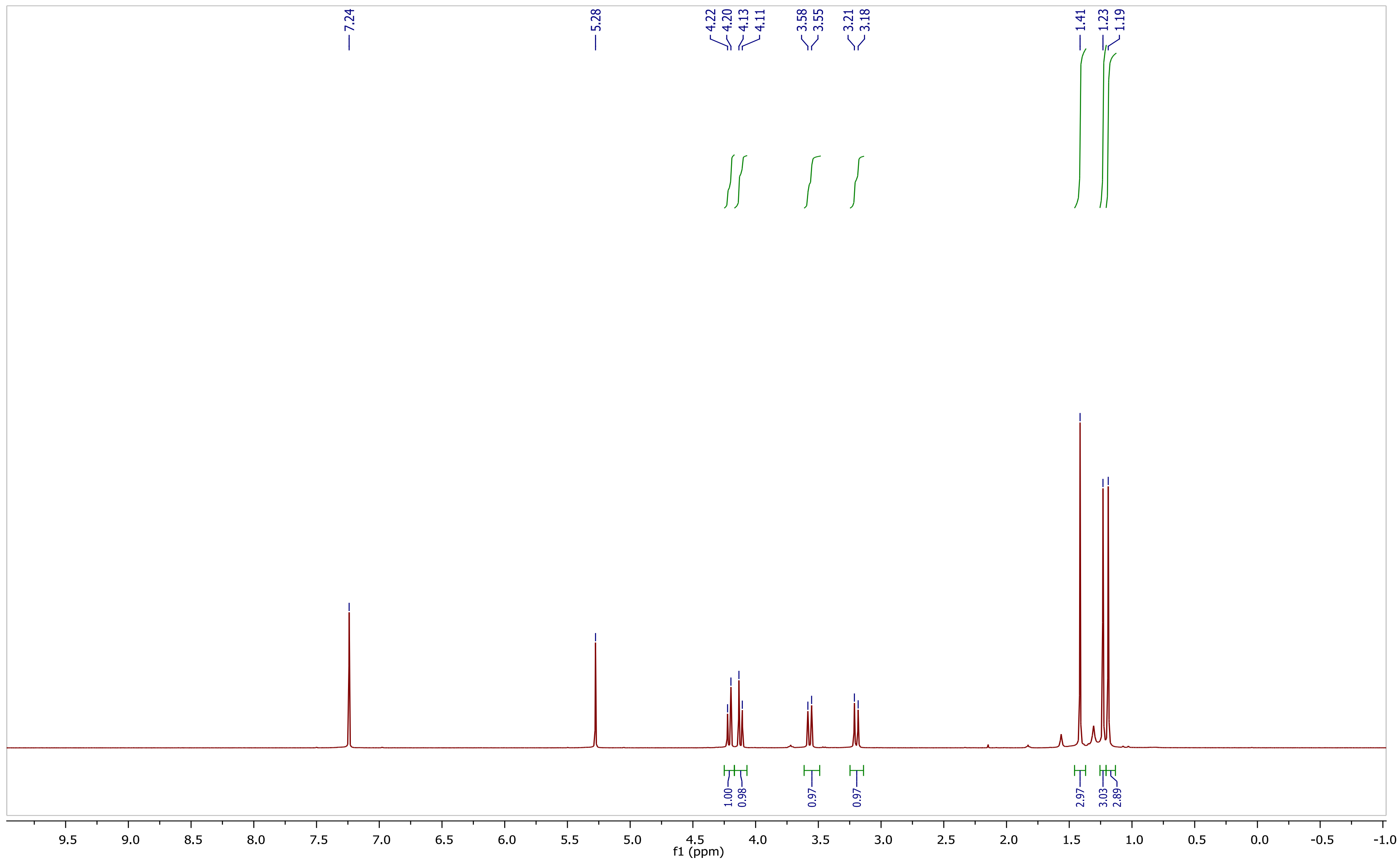

3c 3-(Azidomethyl)-3,5,5-trimethylmorpholin-2-one; $CDCl_3$, 400MHz (^{1}H) 100MHz (^{13}C)

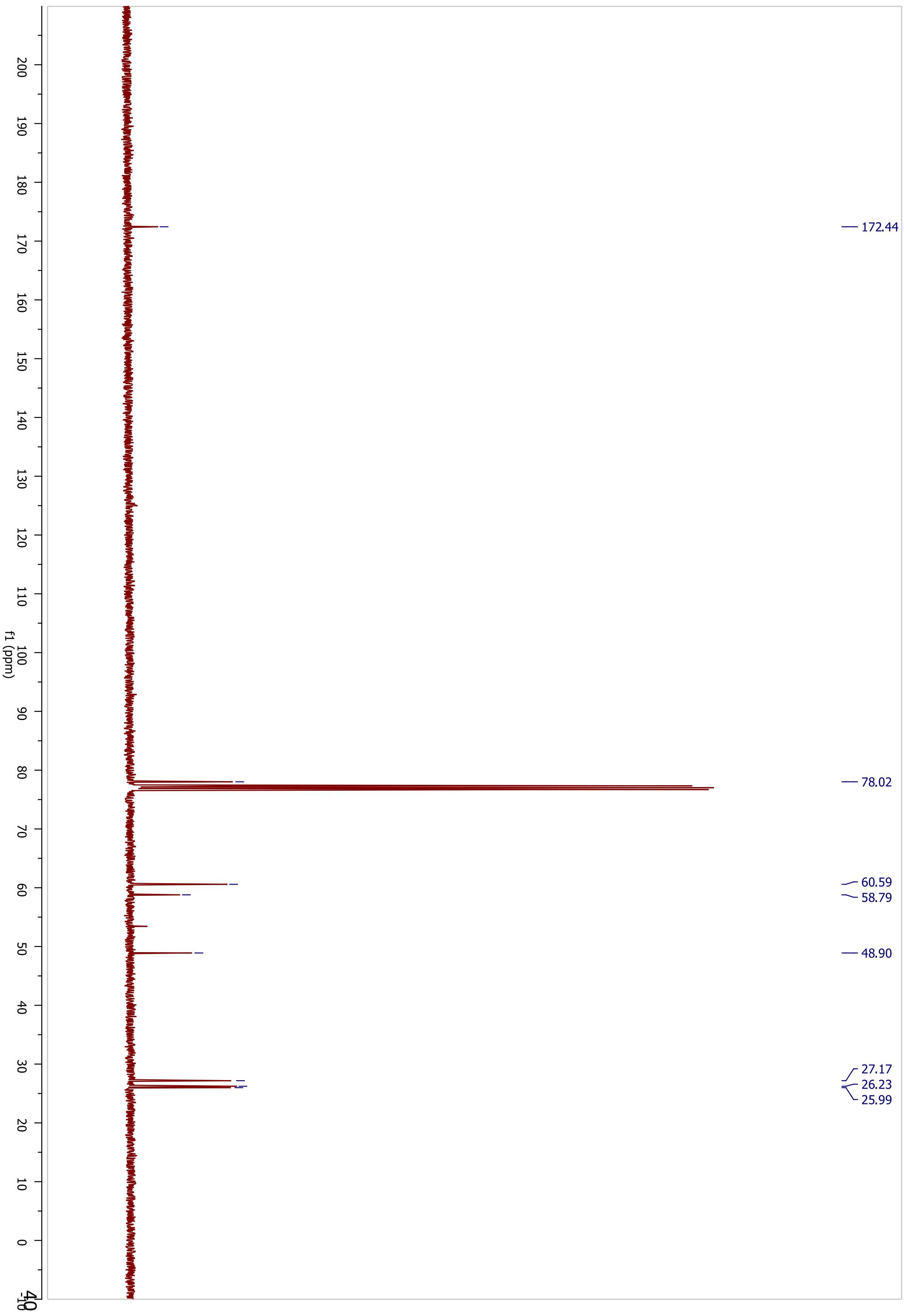
172.44
78.02
60.59
58.79
48.90
27.17
26.23
25.99
f1 (ppm)
